# A biomarker based detection and characterization of carcinomas exploiting two fundamental biophysical mechanisms in mammalian cells

**DOI:** 10.1186/1471-2407-13-569

**Published:** 2013-12-04

**Authors:** Martin Grimm, Steffen Schmitt, Peter Teriete, Thorsten Biegner, Arnulf Stenzl, Jörg Hennenlotter, Hans-Joachim Muhs, Adelheid Munz, Tatjana Nadtotschi, Klemens König, Jörg Sänger, Oliver Feyen, Heiko Hofmann, Siegmar Reinert, Johannes F Coy

**Affiliations:** 1 Department of Oral and Maxillofacial Surgery, University Hospital Tuebingen, Osianderstr. 2-8, 72076, Tuebingen, Germany; 2 German Cancer Research Center (DKFZ) Flow Cytometry Core Facility, Heidelberg, Im Neuenheimer Feld 280, 69120, Heidelberg, Germany; 3 Cancer Research Center, Sanford-Burnham Medical Research Institute, 10901 North Torrey Pines Road, La Jolla, CA 92037, USA; 4 Department of Pathology, University Hospital Tuebingen, Liebermeisterstr. 8, 72076, Tuebingen, Germany; 5 Department of Urology, University Hospital Tuebingen, Hoppe-Seyler-Str. 3, 72076 Tuebingen, Germany; 6 Department of Gynecology, Clemenshospital Muenster, Duesbergweg 124, 48153, Muenster, Germany; 7 Department of Anaesthesiology and Intensive Care Medicine, University Hospital Tuebingen, Hoppe-Seyler-Str. 3, 72076, Tuebingen, Germany; 8 Institute of Pathology, Robert-Koch-Allee 9, 99437, Bad Berka, Germany; 9 TAVARLIN AG, Reißstr. 1a, 64319, Pfungstadt, Germany

**Keywords:** Biomarker, DNaseX, Apo10, TKTL1, EDIM (epitope detection in monocytes), EDIM-blood test, Early detection and diagnosis

## Abstract

**Background:**

Biomarkers allowing the characterization of malignancy and therapy response of oral squamous cell carcinomas (OSCC) or other types of carcinomas are still outstanding. The biochemical suicide molecule endonuclease DNaseX (DNaseI-like 1) has been used to identify the Apo10 protein epitope that marks tumor cells with abnormal apoptosis and proliferation. The transketolase-like protein 1 (TKTL1) represents the enzymatic basis for an anaerobic glucose metabolism even in the presence of oxygen (aerobic glycolysis/Warburg effect), which is concomitant with a more malignant phenotype due to invasive growth/metastasis and resistance to radical and apoptosis inducing therapies.

**Methods:**

Expression of Apo10 and TKTL1 was analysed retrospectively in OSCC specimen (n = 161) by immunohistochemistry. Both markers represent independent markers for poor survival. Furthermore Apo10 and TKTL1 have been used prospectively for epitope detection in monocytes (EDIM)-blood test in patients with OSCC (n = 50), breast cancer (n = 48), prostate cancer (n = 115), and blood donors/controls (n = 74).

**Results:**

Positive Apo10 and TKTL1 expression were associated with recurrence of the tumor. Multivariate analysis demonstrated Apo10 and TKTL1 expression as an independent prognostic factor for reduced tumor-specific survival. Apo10+/TKTL1+ subgroup showed the worst disease-free survival rate in OSCC.

EDIM-Apo10 and EDIM-TKTL1 blood tests allowed a sensitive and specific detection of patients with OSCC, breast cancer and prostate cancer before surgery and in after care. A combined score of Apo10+/TKTL1+ led to a sensitivity of 95.8% and a specificity of 97.3% for the detection of carcinomas independent of the tumor entity.

**Conclusions:**

The combined detection of two independent fundamental biophysical processes by the two biomarkers Apo10 and TKTL1 allows a sensitive and specific detection of neoplasia in a noninvasive and cost-effective way. Further prospective trials are warranted to validate this new concept for the diagnosis of neoplasia and tumor recurrence.

## Background

The immunohistochemical detection of biomarkers in tumor tissue-sections is an essential and powerful technique to determine the malignancy of the tumor and to stratify cancer patient treatment [1]. The success of such stratification strongly depends on the use and quality of biomarkers and their capacity to characterize tumors with regard to malignancy and therapy response. Some biomarkers have already been used for immunohistochemical characterization of tumors. For example, increased proliferation detected by Ki-67 in tumor cells allows a better characterization in terms of malignancy of tumors [2].

In order to establish biomarkers applicable to all tumor entities, biomarkers for two fundamental biophysical mechanisms in mammalian cells have been selected. Despite the extreme complexity of signaling processes within and between cells, only a few principle biophysical mechanisms are known to determine the existence and death of mammalian cells.

One important biophysical mechanism which determines the fate and death of a cell is the cleavage of nuclear DNA by endonucleases [3]. Inhibition of alkaline and acid endonucleases has been identified in tumor cells leading to the suppression of apoptosis [4]. The block of endonuclease activity was due to a factor present in tumor cells [4]. Caspase-activated endonucleases are inhibited by nuclear Akt counteracting apoptosis [5]. Therefore, inhibition of endonuclease (DNase) enzyme activity represents an important biophysical mechanism leading to transformation of healthy cells to tumor cells.

Another important, if not the most important biophysical mechanism of life is the way of energy release within cells. Multicellular organisms depend on energy release either by fermentation or by oxidative phosphorylation (OxPhos). Therefore, only two ways of energy release are possible [6]. While fermentation in eukaryotes is biochemically restricted to sugar metabolites, energy release by oxidation is possible with glucose as well as with amino acids and/or fatty acids [7]. Furthermore, the end product of fermentation (lactic acid) still contains most of the energy. Thus, with regard to energy release OxPhos is superior compared to fermentation. However, despite this, fermentation is the way of choice in cells harboring extremely important DNA like (cancer) stem and germ cells due to safety issues [8]. These cells use this way of energy release to inhibit radical induced DNA damages [8-10], which would lead to DNA mutations in all cells produced by proliferation of stem and germ cells. Cells using OxPhos, which generates fast electrons leading to radical production and DNA damages, do have to pay the price for this efficient, but dangerous way of energy release–they get DNA damages due to radical production [8]. Since radical production is completely prevented by fermentation (substrate chain phosphorylation), stem and germ cells use this way of energy release. Moreover, since fermentation leads to the production of metabolites being able to neutralize (quenching) radicals (e.g. pyruvate, lactic acid), fermentation is also used in cells exposed to a high level of radical production by sun light (retinal cells) or high oxygen concentration (endothelial cells). During evolution of higher vertebrates genome duplication led to duplication of the transketolase (TKT) gene giving rise to the transketolase-like 1 (TKTL1) precursor gene [11,12]. This duplication was followed by an integration of the TKTLI precursor mRNA into the genome creating the intronless and active transketolase-like-2 gene (TKTL2). After this, the TKTL1 precursor gene mutated creating the recent TKTL1 gene. In comparison to the known transketolase proteins, the TKTL1 gene encodes for a TKTL1 protein isoform harboring a 38-amino acid deletion [11,12]. It has been postulated that the altered biochemical properties of the TKTL1 protein(s) represent the basis for a sugar fermentation metabolism linking glucose and fat metabolism independent of pyruvate dehydrogenase [12]. Using metabolic flux analysis and radioactive labeling of sugar metabolites it could be demonstrated that Acetyl-CoA is generated in a TKTL1 dependent way and is incorporated into lipids (Diaz et al., submitted) thus demonstrating a new connection between glucose and lipid metabolism. TKTL1 overexpression has been found in many different cancer types like breast, lung, renal, thyroid, ovarian, colorectal cancer, in tumors of the ocular adnexa and correlates with the increase of metastasis, poor prognosis, tumor recurrence, and resistance to chemo- and radiation therapy [13-28].

The Apo10 protein epitope is detected by the monoclonal antibody Apo10, which has been raised against a DNaseX peptide sequence. DNaseX is a member of the DNaseI-protein family consisting of DNaseI, DNaseX (DNaseI-like 1), DNaseI-like 2 and DNaseI-like 3 (DNase gamma). The Apo10 epitope is present in tumor cells and in very few non-malignant cell types [13,29].

Our study describes the immunohistochemical evaluation of the two biomarkers Apo10 and TKTL1 for characterization of OSCC tissue sections. Furthermore, both biomarkers have been detected intracellularly in monocytes using the epitope detection in monocytes (EDIM) technique, allowing a sensitive and specific noninvasive detection of OSCC, breast and prostate cancer patients by blood samples. This new blood test is based on the EDIM technology [13,29-31], which utilizes the fact that activated monocytes phagocytize and present tumor-related material even in the presence of low tumor mass [32]. Those activated monocytes, which contain intracellular tumor epitopes, can be detected by CD14 and CD16 specific antibodies using flow cytometry [13,29-31].

In the present study, we analysed retrospectively the potential prognostic and predictive influence of Apo10 and TKTL1 expression on clinicopathological parameters and on disease-free survival rates in a large patient cohort with OSCC. In addition to the retrospectively assessed Apo10 and TKTL1 data, prospectively Apo10 and TKTL1 have been determined using the EDIM technique. EDIM-Apo10 and EDIM-TKTL1 blood test was performed in patients with primary and/or recurrent OSCC, breast cancer patients, prostate cancer patients and healthy individuals (blood donors).

## Methods

### Patients and tumor specimen for immunohistochemistry (IHC)

We retrospectively reviewed the records of 161 patients after primary radical R0 tumor resection in our department over a period of ten years and healthy individuals (normal oral mucosa tissues, n = 10). The material had been stored and was investigated with permission of the patients and the local ethics committee (Ethics Committee Tuebingen, Germany, approval number: 001/2012BO2). Patient selection criteria and routine histopathological work-up are described as recently published [33]. Tumor blocks of paraffin-embedded tissue were selected by experienced pathologists, evaluating the routine H.E. stained sections. Sections from all available tumors underwent intensive histopathologic assessment, blinded to the prior histopathology report. Serial tissue sections (2 μm thickness) were cut from formalin-fixed paraffin-embedded (FFPE) blocks on a microtome and mounted from warm water onto adhesive microscope slides. Tumor staging was performed according to the 7th edition of the TNM staging system by the UICC/AJCC of 2010 [34]. Grading was performed according to WHO criteria [35]. Tumor characteristics (UICC stage, pT-categories, pN-categories, cM-categories, infiltrated lymph nodes, residual tumor status, tumor size, site distribution) and patient characteristics (gender, age, personal history, habitual history) were collected in a database (Excel, Microsoft). Tumor and patient characteristics are summarized in Table 1. The mean follow-up was 52.26 months ± 46.21 to 58.31 (95% confidence interval for the mean).

**Table 1:** Clinicopathological characteristics and prognostic factors of 161 patients with OSCC measured by negative and positive Apo10 and TKTL1 expressors

**Characteristics**	**Number of patients**			**p-value**	**Number of patients**		**p-value**
	**Total**	**Apo10 expression**	**Apo10 expression**		**TKTL1 expression**	**TKTL1 expression**	
		**negative (<10%)**	**positive (≥10%)**		**negative (<10%)**	**positive (≥10%)**	
	**n = 161**	**n = 29 (18%)**	**n = 132 (82%)**		**n = 93 (58%)**	**n = 68 (42%)**	
Age (y)				0.5445			0.7507
<60 ± 11.8	80 (49.7%)	16 (20%)	64 (80%)		45 (56%)	35 (44%)	
≥60 ± 11.8	81 (50.3%)	13 (16%)	68 (84%)		48 (59%)	33 (41%)	
Gender				0.8016			0.4501
Male	122 (75.8%)	23 (19%)	99 (81%)		73 (60%)	49 (40%)	
Female	39 (24.2%)	6 (15%)	33 (85%)		20 (51%)	19 (49%)	
Site distribution of OSCC				0.8991			0.8782
Lips	11 (6.8%)	3 (27%)	8 (73%)		9 (82%)	2 (18%)	
Tongue	41 (25.5%)	8 (20%)	33 (80%)		21 (51%)	20 (49%)	
Floor of the mouth	64 (39.8%)	10 (16%)	54 (84%)		37 (58%)	27 (42%)	
Palate	15 (9.3%)	0 (0%)	15 (100%)		6 (40%)	9 (60%)	
Buccal mucosa	8 (5.0%)	3 (38%)	5 (62%)		6 (75%)	2 (15%)	
Alveolar ridge	22 (13.7%)	5 (23%)	17 (77%)		14 (64%)	8 (36%)	
Histological Grading				0.4765*			0.7396*
G1	41 (25.5%)	10 (24%)	31 (76%)		23 (56%)	18 (44%)	
G2	106 (65.8%)	15 (14%)	91 (86%)		63 (59%)	43 (41%)	
G3	13 (8.1%)	3 (23%)	10 (77%)		6 (46%)	7 (54%)	
G4	1 (0.6%)	1 (100%)	0 (0%)		1 (100%)	0 (0%)	
Tumor size				0.0194**			0.0018**
pT1	64 (39.8%)	14 (22%)	50 (78%)		46 (72%)	18 (28%)	
pT2	42 (26.1%)	11 (26%)	31 (74%)		25 (59%)	17 (41%)	
pT3	17 (10.6%)	0 (0%)	17 (100%)		5 (29%)	12 (71%)	
pT4	38 (23.6%)	4 (11%)	34 (89%)		17 (45%)	21 (55%)	
Cervical lymph node metastasis				0.5638			0.0008
pN0	118 (73.3%)	23 (19%)	95 (81%)		78 (66%)	40 (34%)	
pN1-3	43 (26.7%)	6 (14%)	37 (86%)		15 (35%)	28 (65%)	
UICC stage				0.0025***			<0.0001***
UICC I	48 (29.8%)	12 (25%)	36 (75%)		42 (88%)	6 (12%)	
UICC II	36 (22.4%)	11 (31%)	25 (69%)		23 (64%)	13 (36%)	
UICC III	31 (19.3%)	1 (3%)	30 (97%)		11 (35%)	20 (65%)	
UICC IV	46 (28.6%)	5 (11%)	41 (89%)		17 (37%)	29 (63%)	
Locoregional recurrence				0.0125			<0.0001
No	117 (72.7%)	27 (23%)	90 (77%)		81 (69%)	36 (31%)	
Yes	44 (27.3%)	2 (5%)	42 (95%)		12 (27%)	32 (73%)	
Extracapsular spreading of lymph nodes				0.0452			0.0099
No	143 (88,8%)	29 (20%)	114 (80%)		88 (62%)	55 (38%)	
Yes	18 (11,2%)	0 (0%)	18 (100%)		5 (28%)	13 (72%)	
Adjuvant therapy (RTx, Ctx)				0.1202			0.6337
No	111 (68,9%)	24 (22%)	87 (78%)		66 (59%)	45 (41%)	
Yes	50 (31,1%)	5 (5%)	45 (95%)		27 (54%)	23 (46%)	

*Abbrevations:y* years, *G* grading, *UICC* International Union against Cancer, *G1/2 vs. G3/4, **pT1/2 vs. pT3/4, ***UICC I/II vs. UICC III/IV, *RTx* radiotherapy, *CTx* chemotherapy.

### Staining procedure and quantification of IHC

For immunohistochemical analysis, two anti-DNaseX (DeoxyribonucleaseI-like 1, DNaseI-like 1) monoclonal antibodies have been used: Apo10 (TAVARTIS GmbH, Hainburg, Germany, rat anti-human mAb, 5 μg/ml) and ab54750 (abcam, Cambridge, UK, mouse anti-human mAb, 5 μg/ml). Furthermore, monoclonal anti-TKTL1 antibody (TAVARTIS GmbH, Hainburg, Germany, mouse anti-human mAb, 5 μg/ml clone JFC12T10 [12]), and isotype control antibodies (BD Pharmingen, Heidelberg, Germany) were used. The sequence specificity of the Apo10 antibody was demonstrated by preincubation with immunogenic peptide CASLTKKRLDKLELRTEPGF.

Pretreatment and immunohistochemical single staining procedure were performed as described earlier [33]. The secondary antibodies used for immunohistochemical single staining were biotin-conjugated AffiniPure donkey-anti-rat IgG (Apo10) and biotin-conjugated AffiniPure donkey-anti-mouse IgG (TKTL1) used at 1:200 dilution (Jackson ImmunoResearch Laboratories Inc., Suffolk, England).

Five representative chosen high power fields (1 HPF = 0.237 mm^2^) were analysed for Apo10 and TKTL1 expression in the tumor tissue and averaged in each case. The extent of the staining, defined as the percentage of positive staining areas of tumor cells in relation to the whole tissue area, was semi-quantitatively scored on a scale of 0 to 3 as the following: 0, <10%; 1, 10–30%; 2, 30–60%; 3, >60%. The intensities of the signals were scored as 1+, 2+, and 3+. Then, a combined score (0–9) for each specimen was calculated by multiplying the values of these two categories [36]. Cases were classified as: Apo10 and TKTL1 negative, 0 points; Apo10 and TKTL1 positive, 1–9 points. Two observers blinded to the diagnosis performed scoring.

Moreover, for computer-assisted semi-quantitative analysis of TKTL1 expression, ImageJ software (http://rsb.info.nih.gov/ij/) coupled with immunomembrane plug-in (http://153.1.200.58:8080/immunomembrane/) was used to assess the quantification of TKTL1 immunoreactivity in microscopically acquired JPEG images of OSCC samples. Staining completeness (0–10 points) and intensity (0–10 points) were added for a combined score (0–20 points) [37]. Cases were classified as TKTL1 negative, 0 points; TKTL1 positive, 1–20 points. Apo10 expression was analysed by immunoratio plug-in (http://153.1.200.58:8080/immunoratio/). The results were expressed as percentages [38]. From Apo10 and TKTL1 positive slides, 5 images per sample showing representative tumor areas were acquired using 10× and 20× objectives to assess precision (reproducibility/repeatability) of computer-assisted (semi-)quantitative analysis. Pictures were analysed using a Canon camera (Krefeld, Germany). The photographed images were imported into the Microsoft Office Picture Manager.

### Immunohistochemical (IHC) and immunocytochemical (ICC) double staining experiments

The sequential double staining (co-expression) was analysed for Apo10 with TKTL1. The secondary antibody used for IHC/ICC double staining for Apo10 was an alkaline phosphatase (AP)-conjugated AffiniPure donkey-anti-rat IgG (Jackson ImmunoResearch), used at 1:200 dilution. The secondary antibody of TKTL1 was a horseradish peroxidise (HRP)-conjugated AffiniPure donkey-anti-mouse IgG, used at 1:200 dilution (Jackson ImmunoResearch).

For IHC and ICC double staining of Apo10 and TKTL1, slides were incubated with the primary Apo10 antibody or control antibody overnight at 4°C in a humidified chamber and with secondary AP-conjugated antibody for 30 minutes at room temperature in a humidified chamber followed by 20 minutes of incubation with Fast Red (Biogenex, San Ramon, USA). Subsequently, the slides were incubated with the second primary TKTL1 antibody or control antibody overnight at 4°C in a humidified chamber and with secondary HRP-conjugated antibody for 30 minutes at room temperature in a humidified chamber followed by 5 minutes of incubation with 3,3′-Diaminobenzidine (DAB, Biogenex).

Slides were counterstained with Haemalaun and mounted with Glycergel (Dako, Hamburg, Germany). The photographed images were imported into the Microsoft Office Picture Manager.

### Cell culture

We analysed Apo10 and TKTL1 expression in cells (1 × 10^4^) from the OSCC cell lines BICR3 [39], BICR56 [39], and SCC-4 [40] (European Collection of Cell Cultures, ECACC) in cytospins and flow cytometric analysis as a positive control of Apo10 and TKTL1 expression by cancer cells. Preparation and staining of cytospins were performed as described before [33]. BICR3 and BICR56 cells were cultured in DMEM F-12 medium (Invitrogen, Belgium) containing 10% FCS (Sigma-Aldrich, Germany), 1% fungicide, and penicillin/streptomycin (Biochrom, Germany) at 37°C and 5% CO_2_.

### Flow cytometric analysis of OSCC cell lines and EDIM blood tests

Flow cytometric analysis was performed in BICR3, BICR56 cancer cell lines and whole blood as described previously [13,29]. Fluoresceinisothiocyanat (FITC)-conjugated Apo10 and Phycoerythrin (PE)-conjugated TKTL1 antibodies were provided by TAVARLIN AG (Pfungstadt, Germany).

Prospectively, EDIM-TKTL1 and EDIM-Apo10 blood tests were assessed in 50 patients (n = 50) with primary and/or recurrent OSCC as described previously by flow cytometric analysis [13,29]. In these patients, Apo10 and TKTL1 expression had been confirmed by immunohistochemistry of tumor tissue sections. Furthermore, blood samples from 74 healthy blood donors (n = 74, blood donation service, Darmstadt, Germany) were analysed with EDIM-TKTL1 and EDIM-Apo10 blood tests to determine the presence of Apo10 and TKTL1 in CD14/CD16-positive monocytes in the normal population. Furthermore EDIM-TKTL1 and EDIM-Apo10 blood tests have been performed with blood from breast cancer (n = 48) and prostate cancer (n = 115) patients before surgery [13,29]. In all cases, the presence of breast or prostate cancer had been confirmed by analysis of tumor sections by a pathologist.

Informed consent to participate was obtained for the blood tests collected prospectively from patients and volunteers (Ethics Committee Tuebingen, Germany, approval number: 023/2013BO2).

### Determining EDIM scores

Samples were analyzed with a BD FACSCantoII (BD Biosciences, Heidelberg, Germany). At least 10,000 relevant events were collected for each sample. FITC, PE, PerCP and APC signals were recorded as logarithmically amplified data. Analysis was performed using BD FACSDiva software v6.1. (BD Biosciences, Heidelberg, Germany). The result of the EDIM test is given as a relative score indicating the relative amount of CD14/CD16 positive monocytes harbouring Apo10 (or TKTL1) compared to the total amount of CD14/CD16 positive monocytes multiplied with 10. For example, a Apo10 score of 139 means that 13.9% of CD14/CD16 positive monocytes harboured Apo10 intracellularly [13,29]. The results of the EDIM flow cytometric experiments generated by one laboratory (Sven Bellert, Christina Heickenfeld, Melanie Hügen and Oliver Feyen with 14 years of experience in FACS) have been confirmed by an external institute (and independent operator Steffen Schmitt, Head of the Flow Cytometry Unit DKFZ German Cancer Research Centre). The blinded EDIM raw data of ten patients and ten blood donors have been analysed using a different gating strategy independently selected. The obtained results significantly correlated between both analysis methods demonstrating the accuracy of the EDIM results.

### Statistical analysis

Statistical analysis was performed with MedCalc Software, Version 12.7.0 (Mariakerke, Belgium). Disease-free survival (DFS) time was calculated from the time of tumor resection until appearance of obvious locoregional recurrence or tumor conditional death, respectively. The DFS times were estimated using the Kaplan-Meier method [41] and were compared by using the log-rank test [42]. Multivariate analyses were performed using the Cox Proportional Hazards Model [43]. All parameters that were found significant on univariate analysis were included. Hazard ratios (HR) for variables that may influence survival status in univariate and multivariate analysis were provided. Chi-Square test (*χ*^2^) and Fisher’s exact test were used to investigate the relation between two categorical variables. Non-parametric Kendall’s tau (т) correlation coefficient was measured to assess the accuracy (the degree of closeness of measurements of a quantity to that quantity’s actual value) between the two quantification methods of immunohistochemical analysis. All p-values presented were 2-sided and p < 0.05 was considered statistically significant.

To analyse differences in the EDIM-Apo10 and EDIM-TKTL1 scores among healthy individuals (blood donors) and cancer patients, Receiver Operating Characteristics (ROC) analysis was performed [44]. ROC analysis was plotted to determine the best cut off range for healthy individuals compared with cancer group screening EDIM-Apo10 and EDIM-TKTL1 expression to allow a sensitive and specific discrimination between cancer patients and healthy individuals. Area under the curve (AUC) analysis was determined for quality measurement of EDIM-Apo10 and EDIM-TKTL1 expression. The cut-off point was determined as the value corresponding with the highest diagnostic average of sensitivity and specificity (highest diagnostic accuracy). Cut-off points were determined at the score of >109 for EDIM-Apo10, at the score of >117 for EDIM-TKTL1, and at the score of >227 for the combined EDIM-Apo10 and EDIM-TKTL1 expression (summation of both scores) corresponding with highest Youden index. These values were graphical displayed in an Interactive dot diagram to study the accuracy of each diagnostic test.

## Results

### Comparison of observer semi-quantitative scoring with computer-assisted (semi-)quantitative analysis of Apo10 and TKTL1

A preliminary study was carried out to assess the accuracy between the two quantification methods of immunohistochemical analysis. There were significant correlations between the first (observer related semi-quantitative scoring) and the second (computer-assisted (semi)-quantitative analysis) assessment. Apo10 expression: т = 0.868, p < 0.0001 and TKTL1 expression: т = 0.886, p < 0.0001.

### Immunohistochemical analysis of OSCC tumors using two independent anti-DNaseX monoclonal antibodies

Two monoclonal antibodies raised against DNaseX have been selected for expression analysis of OSCC tumors. Antibody ab54750 shows a cytoplasmic and a focal nuclear staining pattern in tumor and in stromal cells, respectively (Additional file 1). Compared to healthy tissue an overexpression in tumor cells can be observed. However, ab54750 staining was detected in human normal oral squamous epithelial cells (n = 6/10 normal oral mucosa samples).

**Figure 1:**
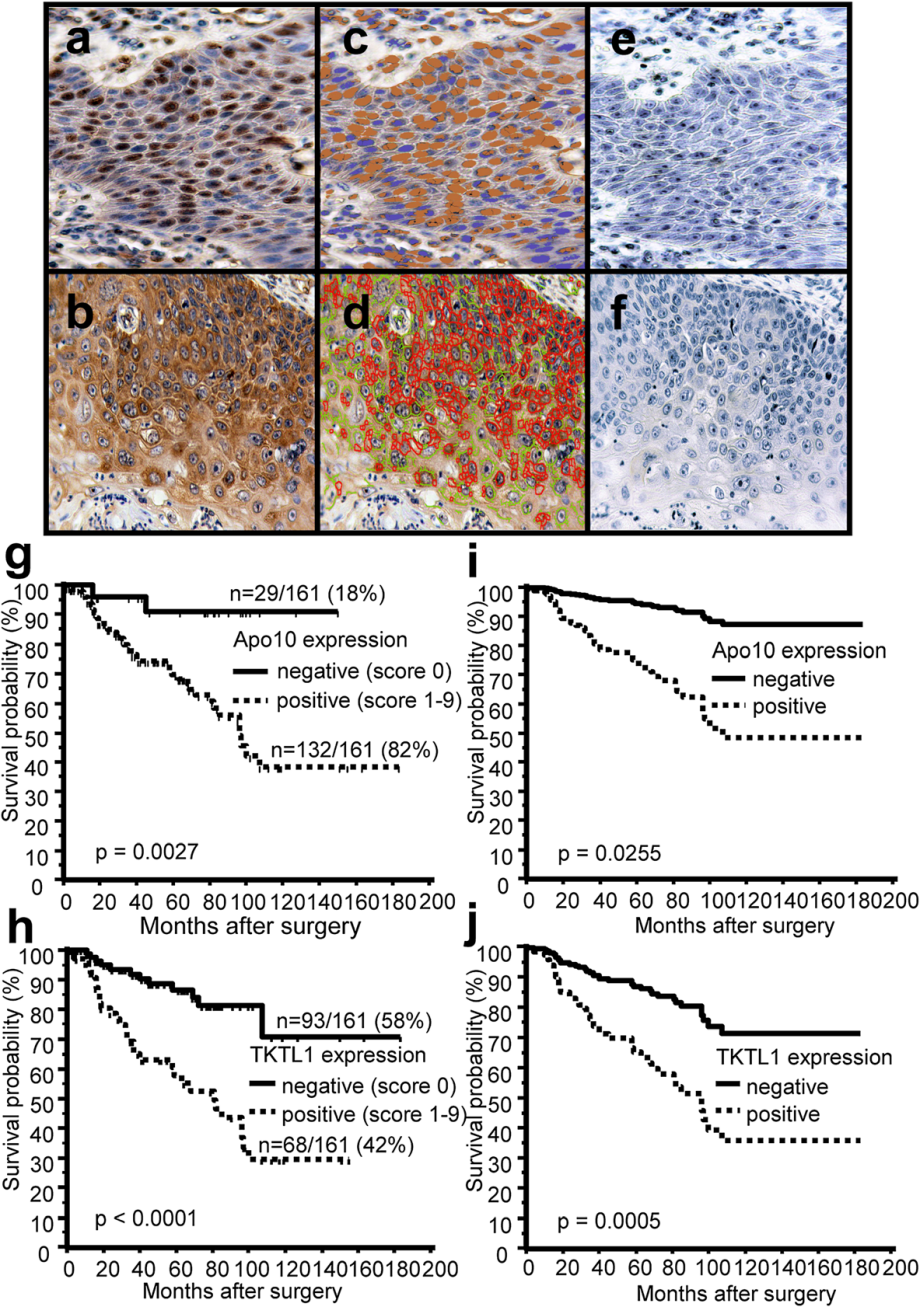
**Immunohistochemical single staining of Apo10, TKTL1 and survival curves of OSCC patients measured by Apo10 and TKTL1 expression.** Brown chromogen color (3,3′-Diaminobenzidine, DAB) indicates positive Apo10 staining (**a**, nuclear staining pattern) and positive TKTL1 expression (**b**, cytoplasmic staining pattern), the blue color shows the nuclear counterstaining by hematoxylin. Pseudo-colored images **(c**, **d)** show the staining components of computer-assisted quantitative analysis in Apo10+ and TKTL1+ tumor cells. Computer-assisted light brown label **(c)** indicates positive Apo10 staining and the computer-assisted light blue label marks the nuclei counterstained with hematoxylin. Computer-assisted red label **(d)** indicates strong or complete TKTL1 staining, the green label **(d)** indicates weak or incomplete staining. Apo10 staining is abolished after incubation with immunogenic peptide **(e)**. Representative image of IgG control **(f)** shows no staining. Original magnification: ×200-fold. Kaplan-Meier (**g**, **h**, left panel) and Cox-regression (**i**, **j**, right panel) survival curves for disease-free survival (DFS) stratified by positive Apo10 and TKTL1 expression (Apo10+, TKTL1+, dashed lines) and negative Apo10, TKTL1 expression (Apo10-, TKTL1-, solid lines). In univariate Kaplan-Meier analysis positive Apo10 **(g)** and TKTL1 **(h)** expression is significantly associated with poorer survival. The times of the censored data are indicated by short vertical lines. Multivariate Cox-regression analysis shows positive Apo10 **(i)** and TKTL1 **(j)** expression as significant independent adverse prognostic factors.

Apo10 (Figure 1) nuclear overexpression was strongly associated with cancer cells and was not detected in stromal or human normal oral squamous epithelial cells (Additional file 2). Apo10 staining was abolished after incubation with the DNaseX peptide, which was used for immunization (Figure 1).

### Apo10 staining in OSCC tumors correlates with tumor size and advanced tumor stages

Using a cut-off of >10% stained cancer cells measured by observer related semi-quantitative scoring, Apo10 expression was detected in 82% of the tumors (n = 132/161). Table 1 shows the clinicopathological characteristics and prognostic factors of 161 OSCC patients with Apo10 negative and Apo10 positive tumors, respectively. Apo10 staining was significantly associated with tumor size (pT3/4, p = 0.0194), advanced tumor stages (UICC III/IV, p < 0.0025), and extracapsular spreading of lymph nodes (p = 0.0452).

### Prognostic value of Apo10 in OSCC

To analyse survival rates in patients after successful (R0) curative surgical resection of OSCC, patients were divided in positive and negative Apo10 expressors (dichotomous variables) measured by observer related semi-quantitative scoring.

In our study population, cervical lymph node metastasis (pN1-3, HR = 2.1145, p = 0.0139) and extracapsular spreading of lymph nodes (p < 0.0001) were shown to be unfavorable factors in univariate analysis of all (n = 161) OSCCs. Tumor size (pT3/4, HR = 1.3865, p = 0.3080) and grading (G3/4, HR = 0.9199, p = 0.8885) were not found to be unfavorable factors in univariate analysis.

To analyse differences in tumor related survival dependent on Apo10 expression in OSCC, we divided the patients in two subgroups as described above. Survival in subgroup with positive Apo10 expression (Apo10+) in OSCCs was significantly worse in comparison to the subgroup of patients lacking Apo10 expression. Apo10+: n = 132, p = 0.0027, HR = 6.4509 (Figure 1). The mean survival for the Apo10- group was 140.24 months ± 125.98 to 154.50 (95% CI for the mean) and for the Apo10+ group 106.40 months ± 89.68 to 123.12 (95% CI for the mean).

Multivariate analysis using the Cox Proportional Hazards Model demonstrated Apo10+ expression as independent prognostic factor in all (n = 161) OSCCs (Table 2, Figure 1).

**Table 2:** Multivariate analysis of prognostic factors of the OSCC study population (n = 161)

**Variable**	**Unfavorable factor**	**Exp (b)**	**95% CI of Exp (b)**	**p-value**
LN	positive	1.4067	0.5357 to 3.6934	0.4950
ECS of lymph nodes	positive	3.1781	1.1567 to 8.7322	0.0257
UICC stage	high (III/IV)	0.5403	0.2323 to 1.2566	0.1550
Apo10	positive	5.1744	1.2317 to 21.7377	0.0255
TKTL1	positive	3.5315	1.7333 to 7.1956	0.0005

LN, Lymph nodes metastasis, Extracapsular spreading (ECS).

### TKTL1 protein is overexpressed in OSCC tumors and correlates with tumor size, advanced tumor stages, and cervical lymph node metastasis

An immunohistochemical analysis of OSCC tumors using anti-TKTL1 monoclonal antibody JFC12T10 was performed. Antibody JFC12T10 has been selected, since it has been shown that monoclonal antibody JFC12T10 specifically detects TKTL1 protein, without cross-reacting with TKT or TKTL2 protein, respectively [12,23]. TKTL1 was not detected in human normal oral squamous epithelial cells (n = 0/10 normal oral mucosa samples). Table 1 shows the clinicopathological characteristics and prognostic factors of 161 OSCC patients with TKTL1 negative and TKTL1 positive tumors, respectively. Applying a cut-off of more than 10% stained tumor cells measured by observer related semi-quantitative scoring (same cut-off was used for Apo10), TKTL1 expression was detected in 42% of the tumors (n = 68/161, Figure 1). TKTL1 expression was significantly associated with tumor size (pT3/4, p = 0.0018), advanced tumor stages (UICC III/IV, p < 0.0001), cervical lymph node metastasis (pN1-3, p = 0.0008), and extracapsular spreading of lymph nodes (p = 0.0099).

### Prognostic value of TKTL1 in OSCC

To analyse survival rates in patients after successful (R0) curative surgical resection of OSCC, patients were divided in positive and negative TKTL1 expressors (dichotomous variables) measured by observer related semi-quantitative scoring.

To analyse differences in tumor related survival dependent on TKTL1 expression in OSCC, we divided the patients in two subgroups as described above. Survival in subgroup with positive TKTL1 expression (TKTL1+) in OSCCs was significantly shorter in comparison to the subgroup of patients lacking TKTL1 expression. TKTL1+: n = 68, p < 0.0001, HR = 3.8382 (Figure 1). The mean survival for the TKTL1- group was 148.80 months ± 129.30 to 168.30 (95% CI for the mean) and for the TKTL1+ group 84.05 months ± 67.20 to 100.91 (95% CI for the mean).

Multivariate analysis using the Cox Proportional Hazards Model demonstrated positive TKTL1 expression (TKTL1+) as independent prognostic factors in all (n = 161) OSCCs (Table 2, Figure 1).

### Prognostic value of Apo10/TKTL1 subgroups in OSCC

Based on Apo10 and TKTL1 expression four subgroups were created. Apo10 was used as a tumor marker for apoptosis inhibition generally present in tumors. TKTL1 expression was used as a metabolic tumor marker indicative of invasion and adverse prognosis. Therefore, subgroups were determined by Apo10-/TKTL1-, Apo10+/TKTL1-, Apo10-/TKTL1+, and Apo10+/TKTL1+ expressors and were associated with clinicopathological characteristics and prognostic factors (Table 3). Compared with Apo10+/TKTL1- (n = 71/161, 44%, red arrow), Apo10+/TKTL1+ subgroup (n = 61/161, 38%) showed the worst DFS (p = 0.0002). The most favorable prognosis was demonstrated for the Apo10-/TKTL1- subgroup (n = 22/161, 14%) (Additional file 3).

**Table 3:** Clinicopathological characteristics and prognostic factors of 161 patients with OSCC measured by Apo10-/TKTL1-, Apo10+/TKTL1-, Apo10-/TKTL1+, and Apo10+/TKTL1+ expressors

**Characteristics**	**Number of Patients**					**p-value**
	**Total**	**Apo10-/TKTL1-**	**Apo10+/TKTL1-**	**Apo10-/TKTL1+**	**Apo10+/TKTL1+**	
	**n = 161**	**n = 22 (14%)**	**n = 71 (44%)**	**n = 7 (4%)**	**n = 61 (38%)**	
Age (y)						0.9067
<60 ± 11.8	80 (49.7%)	13 (16%)	32 (40%)	3 (4%)	32 (40%)	
≥60 ± 11.8	81 (50.3%)	9 (11%)	39 (48%)	4 (5%)	29 (36%)	
Gender						0.3179
Male	122 (75.8%)	18 (15%)	55 (45%)	5 (4%)	44 (36%)	
Female	39 (24.2%)	4 (10%)	16 (41%)	2 (5%)	17 (44%)	
Site distribution of OSCC						0.4899
Lips	11 (6.8%)	3 (27%)	6 (55%)	0 (0%)	2 (18%)	
Tongue	41 (25.5%)	5 (12%)	16 (39%)	3 (7%)	17 (42%)	
Floor of the mouth	64 (39.8%)	7 (11%)	30 (47%)	3 (5%)	24 (37%)	
Palate	15 (9.3%)	0 (0%)	6 (40%)	0 (0%)	9 (60%)	
Buccal mucosa	8 (5.0%)	3 (37.5%)	3 (37.5%)	0 (0%)	2 (25%)	
Alveolar ridge	22 (13.7%)	4 (18%)	10 (45%)	1 (5%)	7 (32%)	
Histological Grading						0.8621*
G1	41 (25.5%)	8 (19%)	15 (37%)	2 (5%)	16 (39%)	
G2	106 (65.8%)	10 (9%)	53 (50%)	5 (5%)	38 (36%)	
G3	13 (8.1%)	3 (23%)	3 (23%)	0 (0%)	7 (54%)	
G4	1 (0.6%)	1 (100%)	0 (0%)	0 (0%)	0 (0%)	
Tumor size						0.0002**
pT1	64 (39.8%)	14 (22%)	32 (50%)	0 (0%)	18 (28%)	
pT2	42 (26.1%)	8 (19%)	17 (41%)	3 (7%)	14 (33%)	
pT3	17 (10.6%)	0 (0%)	5 (29%)	0 (0%)	12 (71%)	
pT4	38 (23.6%)	0 (0%)	17 (45%)	4 (10%)	17 (45%)	
Cervical lymph node metastasis						0.0007
pN0	118 (73.3%)	19 (16%)	59 (50%)	4 (3%)	36 (31%)	
pN1-3	43 (26.7%)	3 (7%)	12 (28%)	3 (7%)	25 (58%)	
UICC stage						<0.0001***
UICC I	48 (29.8%)	12 (25%)	30 (63%)	0 (0%)	6 (12%)	
UICC II	36 (22.4%)	8 (22%)	15 (42%)	3 (8%)	10 (28%)	
UICC III	31 (19.3%)	1 (3%)	10 (32%)	0 (0%)	20 (65%)	
UICC IV	46 (28.6%)	1 (2%)	16 (35%)	4 (9%)	25 (54%)	
Locoregional recurrence						<0.0001
No	117 (72.7%)	21 (18%)	60 (51%)	6 (5%)	30 (26%)	
Yes	44 (27.3%)	1 (2%)	11 (25%)	1 (2%)	31 (71%)	

*Abbrevations:y* years, *G* grading, *UICC* International Union against Cancer, *G1/2 vs. G3/4, **pT1/2 vs. pT3/4, ***UICC I/II vs. UICC III/IV.

### Presence of Apo10 protein epitope in human carcinomas

In order to determine the presence of Apo10 protein epitope in human carcinomas, we performed IHC on 580 human carcinomas derived from 4 different epithelial tumor entities - carcinoma of the lung, colon, (Additional file 4), bladder, and breast (Additional file 5). Similar to the results observed in OSCC, in the majority of carcinomas the Apo10 epitope has been detected.

### Presence of Apo10 protein epitope in benign cells is restricted to very few cell types

In order to determine the presence of Apo10 protein epitope in human benign cells, we performed IHC on 31 samples of myocarditis patients. A nuclear staining was observed and associated with apoptosis rate measured by caspase 3 cleavage (Additional file 4).

### Immunocytochemistry

To determine the expression of Apo10 and TKTL1 in tumor cells, OSCC cell lines BICR3 and BICR56 have been analysed. Single staining of the OSCC cell lines BICR3 and BICR56 in cytospins served as an additional positive control and confirmed the presence of the Apo10 epitope and expression of TKTL1 in cancer cells (Additional file 6).

### Use of flow cytometric analysis for detection of Apo10 and TKTL1 in cancer cells

Flow cytometric analysis confirmed Apo10 and TKTL1 (Additional file 7) labeling in BICR cancer cells as a positive control. To evaluate co-expression of Apo10+/TKTL1+ in cancer cells as suggested by analysis of subgroups in IHC, IHC/ICC double labeling experiments were performed. IHC double staining (representative FFPE OSCC tissue slide, Additional file 8) and ICC double staining of BICR56 (Additional file 7) cancer cells indicates 50–60% TKTL1+ co-expression with Apo10+ in cancer cells.

### EDIM-Apo10 and EDIM-TKTL1 blood tests are highly sensitive and specific for detecting OSCC and recurrence of the tumor

**Figure 2:**
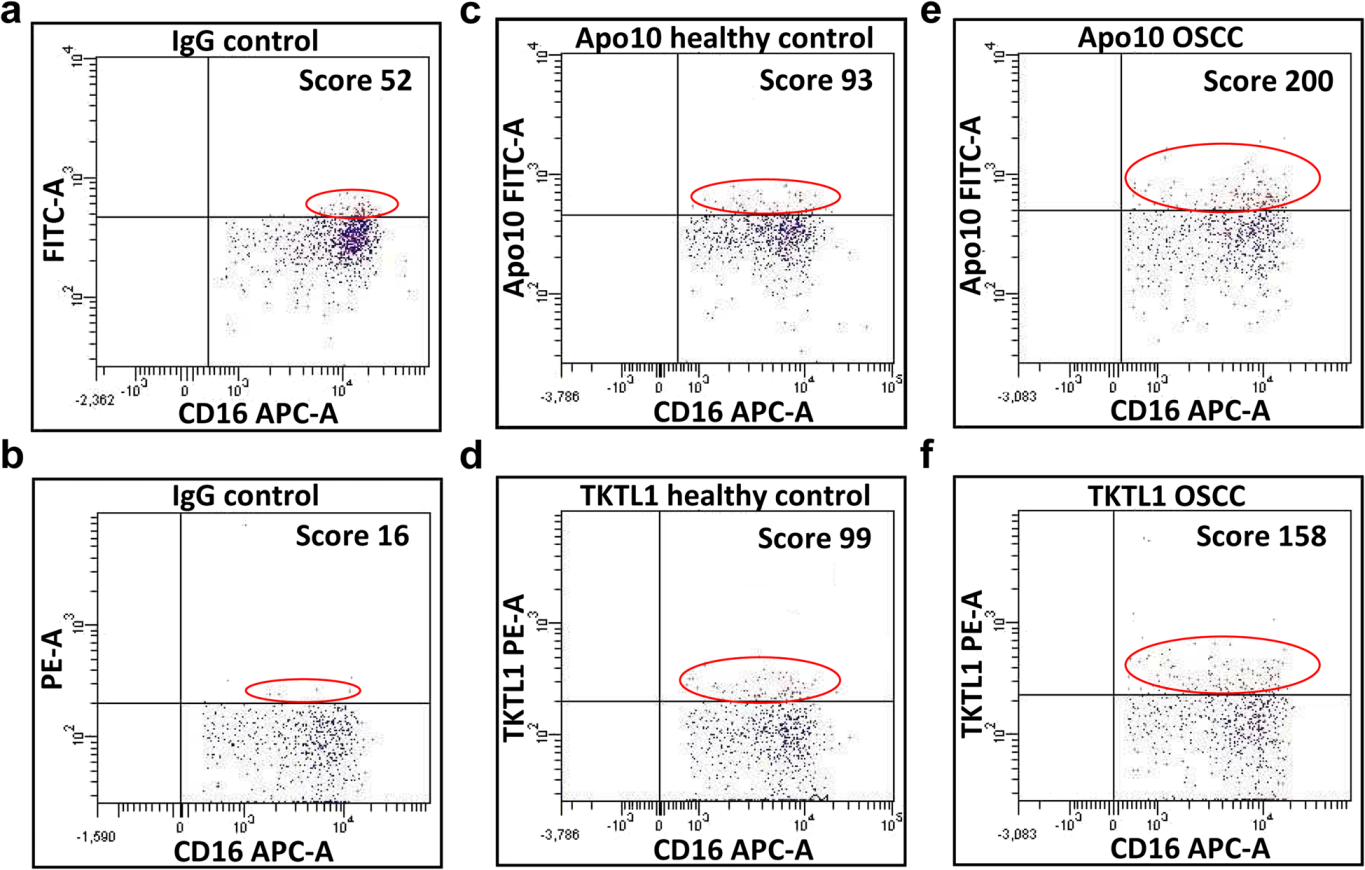
**EDIM-dotplots of Apo10 and TKTL1 staining.** Dotplots show isotype controls (background staining, **a**, Apo10; **b**, TKTL1), healthy control (blood donor, **c**, Apo10; **d**, TKTL1), a patient with OSCC (**e**, Apo10; **f**, TKTL1). Score values indicate the relative amount of positive macrophages. FITC-A (Fluoresceinisothiocyanate area) and PE-A (Phycoerythrin area), red population; APC-A (Allophycocyanin area) blue population.

**Figure 3:**
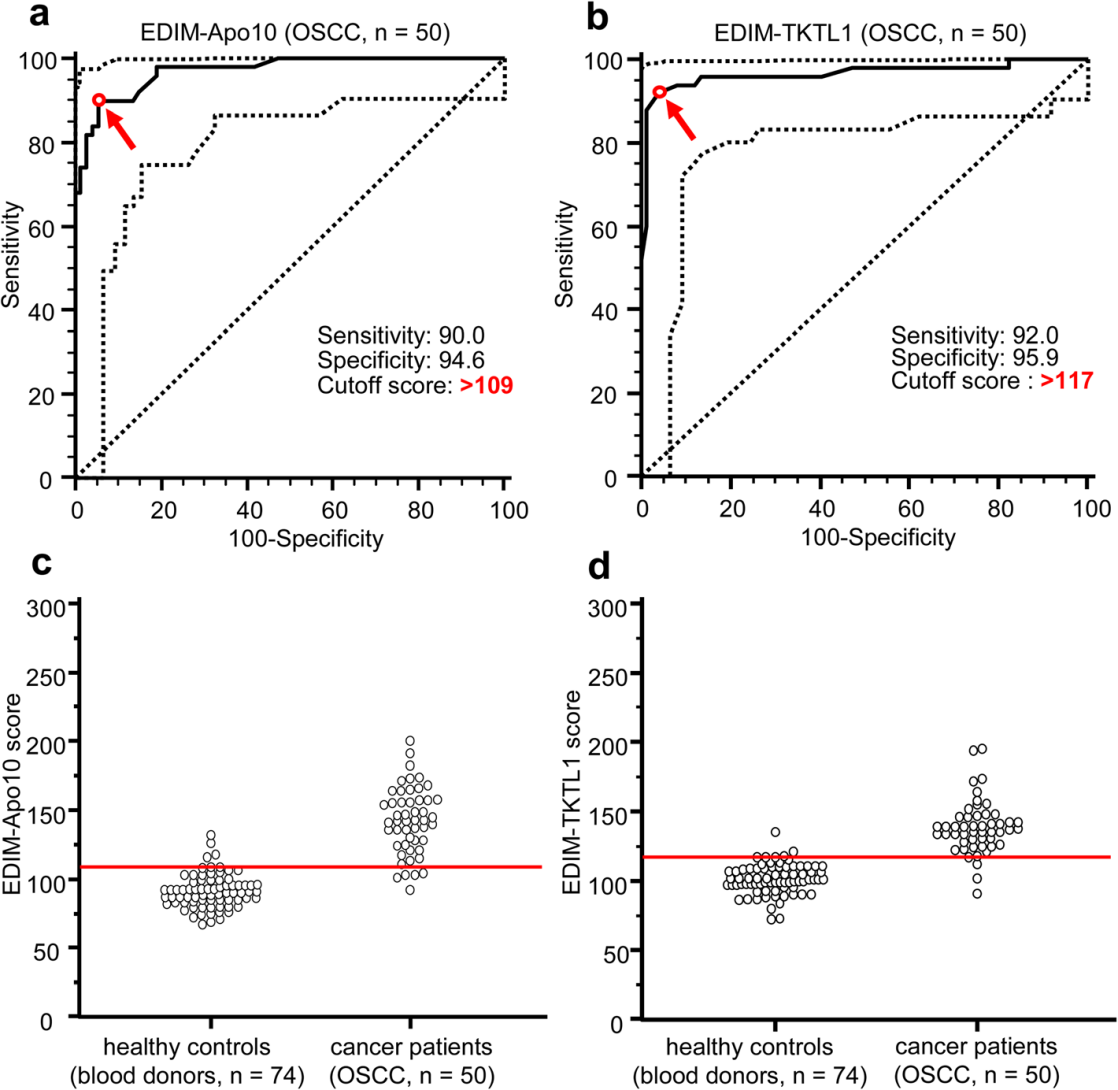
**Receiver Operating Characteristics (ROC) analysis of EDIM-Apo10 and EDIM-TKTL1 score in OSCC (n = 50) compared with healthy individuals (n = 74), and interactive dot diagrams.** The true positive rates (sensitivity) are plotted in function of the false positive rate (100-specificity) for measurement of the cut-off point: ROC analysis for the diagnosis of primary or recurrent OSCC shows calculated cut-off value with highest diagnostic accuracy (arrows) of EDIM-Apo10 **(a)** and EDIM-TKTL1 **(b)** score (**a**, EDIM-Apo10 score >109: sensitivity 90.0%, 95% CI 78.2–96.7%, specificity 94.6%, 95% CI 86.7–98.5%; **b**, EDIM-TKTL1 score >117: sensitivity 92.0%, 95% CI 80.8–97.8%, specificity 95.9%, 95% CI 88.6–99.2%). Dotted lines show 95% CI. OSCC, oral squamous cell carcinoma. In the interactive dot diagrams (part of ROC curve analysis, **c**, **d**) the data of healthy controls and OSCC group are displayed as dots on two vertical axes. The horizontal line indicates the cut-off points with the best separation/highest accuracy (minimal false negative and false positive results) between healthy controls and OSCC group. The corresponding test characteristics sensitivity and specificity are shown above.

Prospectively, EDIM blood tests (Figure 2) were assessed in 50 patients with primary and/or recurrent OSCC. Compared with healthy individuals the ROC-analysis of EDIM-Apo10 (cut-off score >109 EDIM-Apo10 expression: AUC: 0.971, p < 0.0001; Figure 3), EDIM-TKTL1 (cut-off score >117 EDIM-TKTL1 expression: AUC: 0.966, p < 0.0001; Figure 3), and combined EDIM-Apo10 and EDIM-TKTL1 score (cut-off score >227 EDIM-Apo10 plus EDIM-TKTL1 expression: AUC: 0.976, p < 0.0001; Additional file 9) demonstrated a very high sensitivity and specificity for the detection of patients with primary or recurrent OSCC.

45 out of 50 patients with OSCC patients (Table 4) showed positive EDIM-Apo10 scores and 46 patients showed positive EDIM-TKTL1 scores. Only two patients (n = 2/50, 4%) were negative for both values. Using the combined score (EDIM-Apo10/EDIM-TKTL1 >227) 47 OSCC patients were positively detected (Table 4).

**Table 4:** Results of epitope detection in monocytes (EDIM)-blood test in blood donors/controls (n = 74), patients with OSCC (n = 50), breast cancer (n = 48), prostate cancer (n = 115)

	**EDIM-Apo10**		**EDIM-TKTL1**		**EDIM-Apo10/TKTL1**	
	**positive**	**negative**	**positive**	**negative**	**positive**	**negative**
HC (n = 74)	4 (5%)	70 (95%)	3 (4%)	71 (96%)	3 (4%)	71 (96%)
OSCC (n = 50)	45 (90%)	5 (10%)	46 (92%)	4 (8%)	47 (94%)	3 (6%)
BC (n = 48)	42 (88%)	6 (12%)	43 (90%)	7 (10%)	47 (98%)	1 (2%)
PC (n = 115)	109 (95%)	6 (5%)	105 (91%)	10 (9%)	112 (97%)	3 (3%)

HC, healthy controls (blood donors); OSCC, oral squamous cell carcinoma; BC, breast cancer; PC, prostate cancer.

4 out of 74 healthy individuals were positive for EDIM-Apo10, 3 out of 74 individuals were positive for EDIM-TKTL1, and 3 individuals were positive for both values (n = 3/74, 4%). 71 of 74 healthy individuals were negative using the combined score (Table 4).

In 32 out of 50 OSCC patients (n = 32/50), Apo10 and TKTL1 expression had been analysed by immunohistochemistry, since in 18 cases (n = 18/50) biopsy was not possible due to palliative treatment indications in recurrent OSCC or due to biopsy conducted by external surgeons. 29 out of 32 OSCCs were positively stained for Apo10 (n = 29/32, 91%) and 30 out of 32 individuals were positive for TKTL1 (n = 30/32, 94%). Measurement of EDIM-Apo10 and EDIM-TKTL1 revealed normal scoring levels after R0 resection and convalescence (n = 3, Additional file 10: Table S1).

### EDIM-Apo10 and EDIM-TKTL1 blood tests are highly sensitive and specific for detecting patients with breast cancer

EDIM-Apo10 and EDIM-TKTL1 blood tests have been conducted with 48 patients with breast cancer (Table 4) directly before surgery. Only blood samples of patients with histopathologically confirmed breast cancer have been included into the analysis. 42 of 48 patients with breast cancer showed positive EDIM-Apo10 scores and 43 of 48 patients showed positive EDIM-TKTL1 scores (Table 4). All patients were positive for either EDIM-Apo10 or EDIM-TKTL1. The combined score (EDIM-Apo10 plus EDIM-TKTL1) was positive in 47 of 48 breast cancer patients (Table 4). Measurement of EDIM-Apo10 and EDIM-TKTL1 revealed normal scoring levels after R0 resection and convalescence (n = 3, Additional file 11: Table S2).

### EDIM-Apo10 and EDIM-TKTL1 blood tests are highly sensitive and specific for detecting patients with prostate cancer

EDIM-Apo10 and EDIM-TKTL1 blood tests have been conducted with 115 patients with prostate cancer (Table 4) directly before surgery. Only blood samples of patients with histopathologically confirmed prostate cancer have been included into the analysis.

109 of 115 patients with prostate cancer were positive with EDIM-Apo10 blood test and 105 of 115 patients showed positive EDIM-TKTL1 results (Table 4). Only one patient (n = 1/115, 0.87%) was negative for both values. The combined score (EDIM-Apo10 plus EDIM-TKTL1) was positive in 112 of 115 prostate cancer patients (Table 4). Measurement of EDIM-Apo10 and EDIM-TKTL1 revealed normal scoring levels after R0 resection and convalescence (n = 6, Additional file 12: Table S3).

### Comparison of cut-off scores for sensitive and specific detection of patients with OSCC, breast and prostate cancer

ROC-analysis of the EDIM-Apo10, the EDIM-TKTL1, and the combined EDIM-Apo10/EDIM-TKTL1 scores of OSCC, breast and prostate cancer and the three cancer entities together have been compared. Cut-off scores leading to a sensitive and specific detection of breast cancer patients were the same as for a sensitive and specific detection of prostate cancer patients. The same cut-off scores lead to a sensitive and specific detection of OSCC, breast and prostate cancer patients (Additional file 13).

### Patterns of treatment

Most patients underwent surgery (69%, n = 111) alone as definitive therapy, whereas 50 (31%) patients had adjuvant radiotherapy with/without chemotherapy. Adjuvant treatment in association with UICC stages is shown in Additional file 14: Table S4. The association of the adjuvant treatment with Apo10 and TKTL1 expression results is given in Table 1.

## Discussion

In 1991 Nobel laureate Professor Harald zur Hausen initiated a genome analysis program in the German Cancer Center focusing on the genomic region Xq28. At that time there was no evidence for the presence of cancer related genes in this region except the already known MAGE genes. This genomic region was systematically cloned in cosmids, ordered in contigs and further analysed for the presence of genes, conserved between human and pig, encoding tissue-specific expressed transcripts [11]. As a result of this approach the DNaseX and TKTL1 gene have been identified [11,12]. Both genes represent the result of a genome duplication event leading to a copy of a DNaseI and transketolase (TKT) precursor gene, each. Gene duplications played an important role in the evolution of higher vertebrates, since the gene copies allowed an evolution of the function and regulation of genes without destroying the primary function of the gene which was copied [11]. DeoxyribonucleaseI as well as transketolase represent highly conserved enzymes which were the target of gene duplications leading to new DNase and transketolase genes/proteins, with sophisticated changes of expression and function of the copied genes. Those altered functions had implications for the evolution of higher vertebrates due to better adaptions of cells and multicellular organisms, but may also have implications for arising and development of nonmalignant and malignant tumor cells.

To analyse the expression of DNaseX protein in OSCC, a DNaseX peptid has been used to generate monoclonal antibody Apo10. Although another commercially available anti-DNaseX monoclonal antibody revealed an overexpression of the detected protein in OSCC, its staining pattern is distinct from Apo10 antibody and less tumor specific. This difference in staining patterns of both antibodies could be due to differences in crossreactivity to other DNaseI protein family members or even unrelated proteins, but could also be the consequence of epitope masking by protein binding. Such selective epitope detection by monoclonal antibodies has been revealed for DNase-gamma (DNaseI-like 3) [45]. Two monoclonal antibodies have been raised against a DNase-gamma specific peptide. Whereas one antibody detected a constitutively expressed DNase-gamma protein variant, the other monoclonal antibody specifically detected a nuclear DNase-gamma protein variant as a consequence of apoptosis induction by X-ray radiation, suggesting that some molecular change(s), which triggers the activation of DNase-gamma, occurs in response to apoptotic stimuli in the detected protein domain [45].

Similar to the DNase-gamma peptide, the DNaseX peptide may represent an epitope (Apo10 epitope) which is the target of molecular and/or biochemical change(s) leading to differential accession by monoclonal antibodies. Monoclonal antibody Apo10 detects a protein epitope present in the nucleus of apoptotic benign cells, but also in the nucleus of tumor cells. Whereas benign apoptotic cells (e.g. heart muscle cells of myocarditis patients) execute apoptosis, tumor cells apparently block the endonuclease driven execution of apoptosis by expression of proteins inhibiting endonucleases [4,5] or other apoptosis executing proteins. Although the mechanism of activation and blocking of apoptosis is not fully understood, the presence of endonuclease epitopes in the nucleus can be exploited for tumor cell detection.

The Apo10 epitope is present in neoplastic cells including carcinomas, sarcomas, glioblastomas, lymphomas, and leukemias [29], whereas only few benign cells with and without induction of apoptosis show this epitope. The presence of the Apo10 epitope in OSCC and other neoplasias is a widespread event. Apo10 protein was present in 82% of primary OSCC tumors and correlated with poor patient prognosis.

Although the overexpression of TKTL1 takes also place in carcinomas [16], sarcomas [13], glioblastomas [26], lymphomas [27], and leukemias, the percentage of tumors overexpressing TKTL1 is lower compared to Apo10. 42% of the 161 tested primary OSCC showed an overexpression of TKTL1. Almost all TKTL1 positive tumors turned out to be Apo10 positive, whereas only 4% of the 161 tested primary OSCC were Apo10-/TKTL1+. This indicates that Apo10 precedes TKTL1 activation. This is in line with the currently accepted cancer theory that arising of tumor cells is based on the acquisition of mutations in different genes controlling cellular processes like apoptosis and metabolism. By using the biomarkers Apo10 and TKTL1 it is now possible to detect neoplasia associated changes in the two fundamental biophysical processes of endonuclease/apoptosis activation and glucose/energy metabolism.

The clinical relevance of these two fundamental biophysical processes is underlined by the fact, that Apo10 and TKTL1 presence in tumors represent two independent processes which are associated with advanced tumor stages and reduced tumor-specific survival in OSCC. This allows for the first time a two-step model of detection and characterization of tumors based on two biomarkers. OSCC tumors negative for Apo10 and TKTL1 represented tumors with favourably prognostic impact on survival, Apo10 presence indicated malignant OSCC tumors, and Apo10+/TKTL1+ OSCC tumors turned out to be more malignant tumors with an invasive/metastasing phenotype and worst prognostic impact on survival.

A large number of studies underlined the clinical relevance of increased TKTL1 expression on gene, transcript and protein level, since TKTL1 expression correlates with poor patient outcome and metastasis in many solid tumors [14-27]. Inhibition of TKTL1 mRNA translation has been shown to inhibit cancer cell proliferation and to decrease lactate production [14,15]. Specifically in head and neck squamous cell carcinoma TKTL1 is activated by promoter hypomethylation and drives carcinogenesis by increased aerobic glycolysis and hypoxia inducible factor 1 alpha (HIF-1α) stabilization [19]. A more recently published study describes TKTL1 to be indispensable for the function of the p53-dependent effector Tp53-induced glycolysis and apoptosis regulator (TIGAR) on hypoxia-induced cell death. Besides their diagnostic opportunity for cancer detection, these data confirm TKTL1 as an important new target for cancer treatment allowing inhibition of tumor cell viability and the increase of sensitivity towards hypoxia-, apoptosis and reactive oxygen species inducing therapies [15,19,24,46].

A multistep process comprising initially six biological capabilities has been proposed as the basis for development of human tumors. The so called hallmarks of cancer include sustaining proliferative signaling, evading growth suppressors, resisting cell death, enabling replicative immortality, inducing angiogenesis, and activating invasion and metastasis [47]. Our Apo10 and TKTL1 results contribute to the hallmarks of abnormal apoptosis/proliferation and increased invasion and metastasis, respectively. In addition to these intrinsic characteristics of tumor cells leading to tumor growth, the immune system based elimination of tumor cells determines whether a net gain of tumors happens or not. Therefore, a prerequisite of tumor growth is the outbalance of growth compared to the elimination of tumor cells. The hallmarks of cancer not only influence the growth rate of tumors but also influence the elimination rate by the immune system. The presence of tumor specific antigens facilitates the detection and elimination of tumor cells either by cytotoxic killing of tumor cells or by phagocytosis of tumor cells executed by macrophages. The elimination of tumor cells by cytotoxic killing of tumor cells is strongly dependent on tumor metabolism, since fermentation of glucose to lactic acid even in the presence of oxygen (aerobic glycolysis/Warburg effect [48]) prevents killing of tumor cells by natural killer cells [49]. Furthermore lactic acid excretion by tumor cells allows an acid based degradation of surrounding matrix of healthy tissue leading to invasive growth. Matrix degradation in distant organs allows disseminated tumor cells to build distant colonies thereby leading to metastases [6,7]. Therefore, the metabolic switch from a mitochondria-based energy metabolism (OxPhos) to glucose fermentation e.g. mediated by TKTL1 is the basis of an invasive and metastasis inducing malignant phenotype of tumors as well as the basis of an immune protective strategy avoiding elimination of tumor cells by natural killer cells or cytotoxic T cells. Furthermore, since the metabolic switch from a mitochondria-based energy release to fermentation inhibits apoptosis induction (via reducing cytochrome c) and radical induction, elimination of tumor cells by apoptosis and radical inducing therapies (e.g. chemotherapy, radiation, respectively) is suppressed by TKTL1 metabolism [7,15,19,24,46].

In a proof of concept study the biomarkers Apo10 and TKTL1 have been used exploiting the epitope detection in monocytes (EDIM) technology. The EDIM technology allows a noninvasive detection of tumor proteins in blood. EDIM-Apo10 and EDIM-TKTL1 blood tests have been prospectively conducted in patients with primary or recurrent OSCC as well as in patients with primary breast and prostate cancer. In patients with histologically confirmed OSCC, breast and prostate cancers, blood samples before surgery revealed significant elevated levels of Apo10 and TKTL1 in CD14/CD16 positive monocytes compared to blood donors. By using a single cut-off for all three tumor entities it was possible to identify cancer patients by either EDIM-Apo10 or EDIM-TKTL1 blood test in a specific and sensitive manner. The combination of EDIM-TKTL1 and EDIM-Apo10 scores increased the sensitivity to 95.8% and the specificity to 97.3% in all cancer samples/entities.

## Conclusions

The combined detection of two independent fundamental biophysical processes by the two biomarkers Apo10 and TKTL1 may allow a sensitive and specific detection of neoplasia in a noninvasive and cost-effective way. Further prospective trials are warranted to validate this new concept for the diagnosis of neoplasia and tumor recurrence.

## Competing interests

OF, HH and JFC are employees and shareholders of TAVARLIN AG, Pfungstadt, Germany and declare a potential conflict of interest due to the possible utilization of Apo10/DNaseX and TKTL1 for diagnostic and/or therapeutic purposes. The authors have no other affiliations or financial involvement with any organization or entity with a financial interest in or financial conflict with the subject matter or materials discussed in the manuscript apart from those disclosed.

## Authors’ contributions

MG and JFC conceived the study, carried out immunohistochemistry studies, performed the statistical analyses, and drafted the manuscript. OF, SS and PT performed flow cytometric analysis. TB and JS analysed histopathological specimen and carried out immunohistochemistry studies. AS, JH, HJM, and KK carried out the data collection. AM and TN performed cell culture experiments. OF, HH, and SR participated in the design of the study and coordination and drafted the manuscript. All authors read and approved the final manuscript.

## Pre-publication history

The pre-publication history for this paper can be accessed here:

http://www.biomedcentral.com/1471-2407/13/569/prepub

## Supplementary Material

Additional file 1 **DNaseX staining.** Immunohistochemistry shows representative images of antibody ab54750 staining **(a, b)** compared to Apo10 staining **(c, d)**. Antibody ab54750 shows cytoplasmic and a focal nuclear staining pattern, whereas Apo10 is detected exclusively in the nucleus. The blue color shows the nuclear counterstaining by hematoxylin. The square box demonstrates the area of interest (original magnification: ×100-fold, upper panel) which is also shown in larger magnification (×200-fold, lower panel). Click here for file

Additional file 2 **DNaseX (Apo10) staining in human normal oral squamous epithelial cells.** Immunohistochemistry shows representative image of Apo10 staining in human normal oral squamous epithelial cells. Apo10 is not detected in human normal oral squamous epithelial cells. The blue color shows the nuclear counterstaining by hematoxylin. Original magnification: ×200-fold. Click here for file

Additional file 3 **Survival curve of OSCC patient subgroup analysis measured by Apo10/TKTL1 co-expression.** Kaplan-Meier survival curves for DFS stratified by Apo10-/TKTL1- (blue line), Apo10+/TKTL1- (red line), Apo10-/TKTL1+ (grey line), and Apo10+/TKTL1+ (green line) subgroups (a). Compared with Apo10+/TKTL1- (red line), Apo10+/TKTL1+ (green line) subgroup shows the worst DFS (red arrow, p = 0.0002). The most favorable prognosis is demonstrated by the Apo10-/TKTL1- (blue line) subgroup. Click here for file

Additional file 4 **DNaseX (Apo10) staining in benign cells of the myocardium and two different human epithelial tumor entities - carcinomas of the lung, and colon.** Hematoxylin and eosin stain (H&E) shows myocardium **(a)** and different types of carcinomas **(b, c, d)**. Immunohistochemistry shows representative images of Apo10 (**e, f, g, h,** arrows) in human apoptotic (Caspase-3 cleaved, **i, j, k, l,** arrows) benign cells of a patient after myocarditis and in carcinomas of the lung, and colon, which is detected in the nucleus. Apoptotic cells (Caspase-3 cleaved) are increased in benign tissue **(i)** compared with decreased detection of apoptotic cells in carcinomas **(j, k, l)**. Both, benign and malign tissue types stained Apo10+. The blue color shows the nuclear counterstaining by hematoxylin. Original magnification: ×200-fold. AC, adenocarcinoma; SCC, Squamous cell carcinoma. Click here for file

Additional file 5 **DNaseX (Apo10) staining in bladder and breast carcinoma.** Hematoxylin and eosin stain (H&E) shows bladder and breast carcinomas **(a, b)**. Immunohistochemistry shows representative images of Apo10 (**c, d,** arrows) in human apoptotic (Caspase-3 cleaved, **e, f,** arrows) cells in carcinomas of the bladder and mammary gland (breast), which is detected in the nucleus. Apoptotic cells (Caspase-3 cleaved) in carcinomas **(e, f)** are decreased compared with benign tissue (myocardium, Additional file 4). Both, benign and malign tissue types stained Apo10+. The blue color shows the nuclear counterstaining by hematoxylin. Original magnification: ×200-fold. AC, adenocarcinoma. Click here for file

Additional file 6 **DNaseX (Apo10) and TKTL1 immunocytochemical staining in BICR3, BICR56, and SCC-4 OSCC cell lines.** IgG control shows no staining **(a, b, c)**. Images show representative immunocytochemical staining of Apo10 (nuclear and weak cytoplasmic expression pattern, **d, e, f**), and TKTL1 (cytoplasmic staining expression pattern, **g, h, i**). The blue color shows the nuclear counterstaining by hematoxylin. Original magnification: ×400-fold. Click here for file

Additional file 7 **Flow cytometric analysis of Apo10+, TKTL1+ cancer cells and immunocytochemical Apo10+/TKTL1+ double staining.** Flow cytometric analysis shows representative Apo10 **(a)** and TKTL1 **(b)** labeling in BICR56 cancer cells as a positive control. FITC, Fluoresceinisothiocyanate. Immunocytochemical staining shows a representative image of Apo10+/TKTL1+ **(c)** tumor cells in BICR56 OSCC cell line. The red nuclear chromogen color (Fast Red) indicates positive Apo10 staining and the brown cytoplasmic chromogen color (DAB) indicates positive TKTL1 staining (arrows). Asterisks show single Apo10 (red) or TKTL1 (brown) positive cells. Original magnification: ×400-fold. Click here for file

Additional file 8 **Immunohistochemical Apo10+/TKTL1+ double staining.** Immunohistochemical Apo10+/TKTL1+ double staining of a representative double positive OSCC tissue shows nuclear Apo10+ (red arrow, Fast Red) and cytoplasmic TKTL1+ (brown arrow, DAB) co-expression. The square box demonstrates area of interest (original magnification: ×100-fold, **a)**, which is also shown in a larger magnification (×200-fold, lower panel, **b)**. Click here for file

Additional file 9 **Receiver Operating Characteristics (ROC) analysis of combined EDIM Apo10/TKTL1 score in OSCC (n = 50) compared with healthy individuals (n = 74), and interactive dot diagrams.** The true positive rates (sensitivity) are plotted in function of the false positive rate (100-specificity) for measurement of the cut-off point: ROC analysis for the diagnosis of primary or recurrent OSCC shows calculated cut-off value with highest diagnostic accuracy (arrows) of combined EDIM Apo10/TKTL1 **(a)** score combined EDIM-Apo10 plus EDIM-TKTL1 score >227: sensitivity 94.0%, 95% CI 83.5–98.7%, specificity 97.3%, 95% CI 90.6–99.7%). Dotted lines show 95% CI. OSCC, oral squamous cell carcinoma. In the interactive dot diagrams (part of ROC curve analysis, **b)** the data of healthy controls and OSCC group are displayed as dots on two vertical axes. The horizontal line indicates the cut-off points with the best separation/highest accuracy (minimal false negative and false positive results) between healthy controls and OSCC group. The corresponding test characteristics sensitivity and specificity are shown above. Click here for file

Additional file 10: Table S1 Pre- and postoperative epitope detection in monocytes (EDIM)-Apo10 and TKTL1 scores in patients with oral squamous cell carcinoma (n = 3). Click here for file

Additional file 11: Table S2 Pre- and postoperative epitope detection in monocytes (EDIM)-Apo10 and TKTL1 scores in patients with breast cancer (n = 3). Click here for file

Additional file 12: Table S3 Pre- and postoperative epitope detection in monocytes (EDIM)-Apo10 and TKTL1 scores in patients with prostate cancer (n = 6). Click here for file

Additional file 13 **Receiver Operating Characteristics (ROC) analysis of EDIM-Apo10, EDIM-TKTL1, and combined EDIM Apo10/TKTL1 score in all cancer samples (OSCC, breast and prostate cancer, n = 213) compared with healthy individuals (n = 74).** The true positive rates (sensitivity) are plotted in function of the false positive rate (100-specificity) for measurement of the cut-off point: ROC analysis for the diagnosis of all cancer samples/entities (OSCC, breast and prostate cancer, a-c) shows calculated cut-off value with highest diagnostic accuracy (arrows) of EDIM-Apo10 **(a)**, EDIM-TKTL1 **(b)**, and combined EDIM Apo10/TKTL1 **(c)** score **(a**, EDIM-Apo10 score >109: sensitivity 92.0%, 95% CI 87.5–95.3%, specificity 94.6%, 95% CI 86.7–98.5%; **b,** EDIM-TKTL1 score >117: sensitivity 90.6%, 95% CI 85.9–94.2%, specificity 95.9%, 95% CI 88.6–99.2%; **c,** combined EDIM-Apo10 plus EDIM-TKTL1 score >227: sensitivity 95.8%, 95% CI 92.1–98.0%, specificity 97.3%, 95% CI 90.6–99.7%). Dotted lines show 95% CI. OSCC, oral squamous cell carcinoma; BC, breast cancer; PC, prostate cancer. In the interactive dot diagrams (part of ROC curve analysis, **d-f**) the data of healthy controls and cancer group are displayed as dots on two vertical axes. The horizontal line indicates the cut-off points with the best separation/highest accuracy (minimal false negative and false positive results) between healthy controls and cancer group. The corresponding test characteristics sensitivity and specificity are shown above. Click here for file

Additional file 14: Table S4 Adjuvant treatment of 161 patients with OSCC according to UICC stages. Click here for file
