# Supplementary figures and images for: A biomarker based detection and characterization of carcinomas exploiting two fundamental biophysical mechanisms in mammalian cells
Source: BMC Cancer. 2013 Dec 4;13:569. doi: 10.1186/1471-2407-13-569 (PMC4235042; doi:10.1186/1471-2407-13-569)

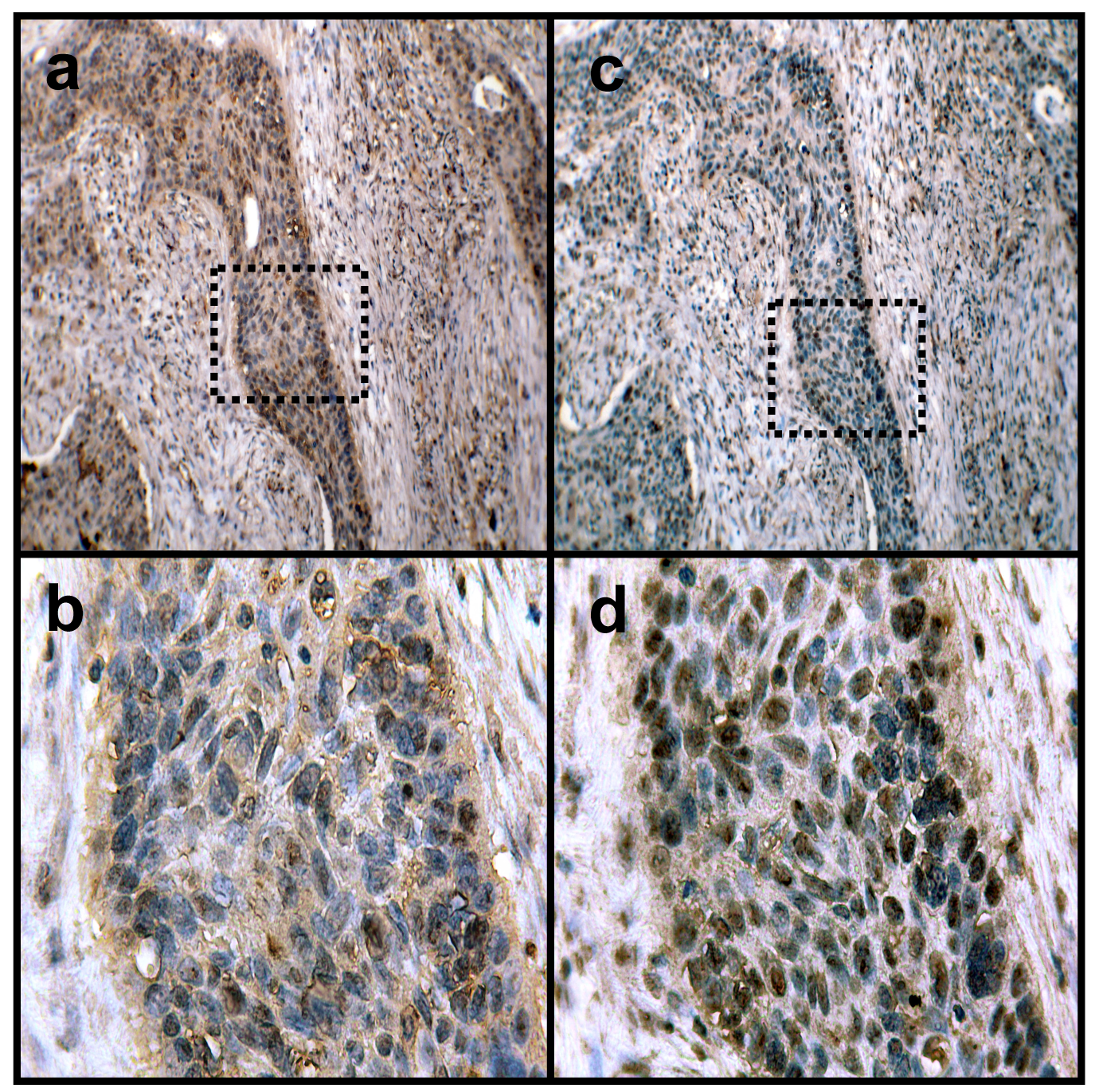

Supplement: Additional file 1 — DNaseX staining. Immunohistochemistry shows representative images of antibody ab54750 staining (a, b) compared to Apo10 staining (c, d). Antibody ab54750 shows cytoplasmic and a focal nuclear staining pattern, whereas Apo10 is detected exclusively in the nucleus. The blue color shows the nuclear counterstaining by hematoxylin. The square box demonstrates the area of interest (original magnification: ×100-fold, upper panel) which is also shown in larger magnification (×200-fold, lower panel). [file 1471-2407-13-569-S1.tiff]

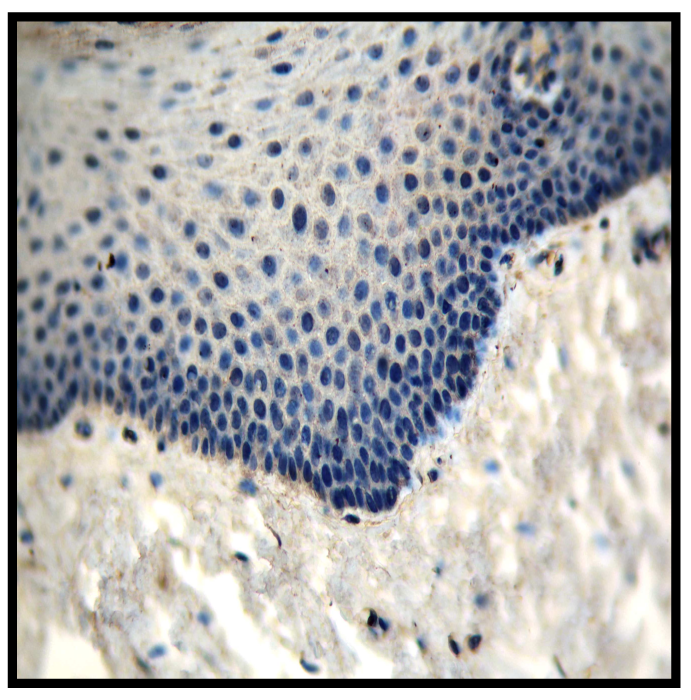

Supplement: Additional file 2 — DNaseX (Apo10) staining in human normal oral squamous epithelial cells. Immunohistochemistry shows representative image of Apo10 staining in human normal oral squamous epithelial cells. Apo10 is not detected in human normal oral squamous epithelial cells. The blue color shows the nuclear counterstaining by hematoxylin. Original magnification: ×200-fold. [file 1471-2407-13-569-S2.tiff]

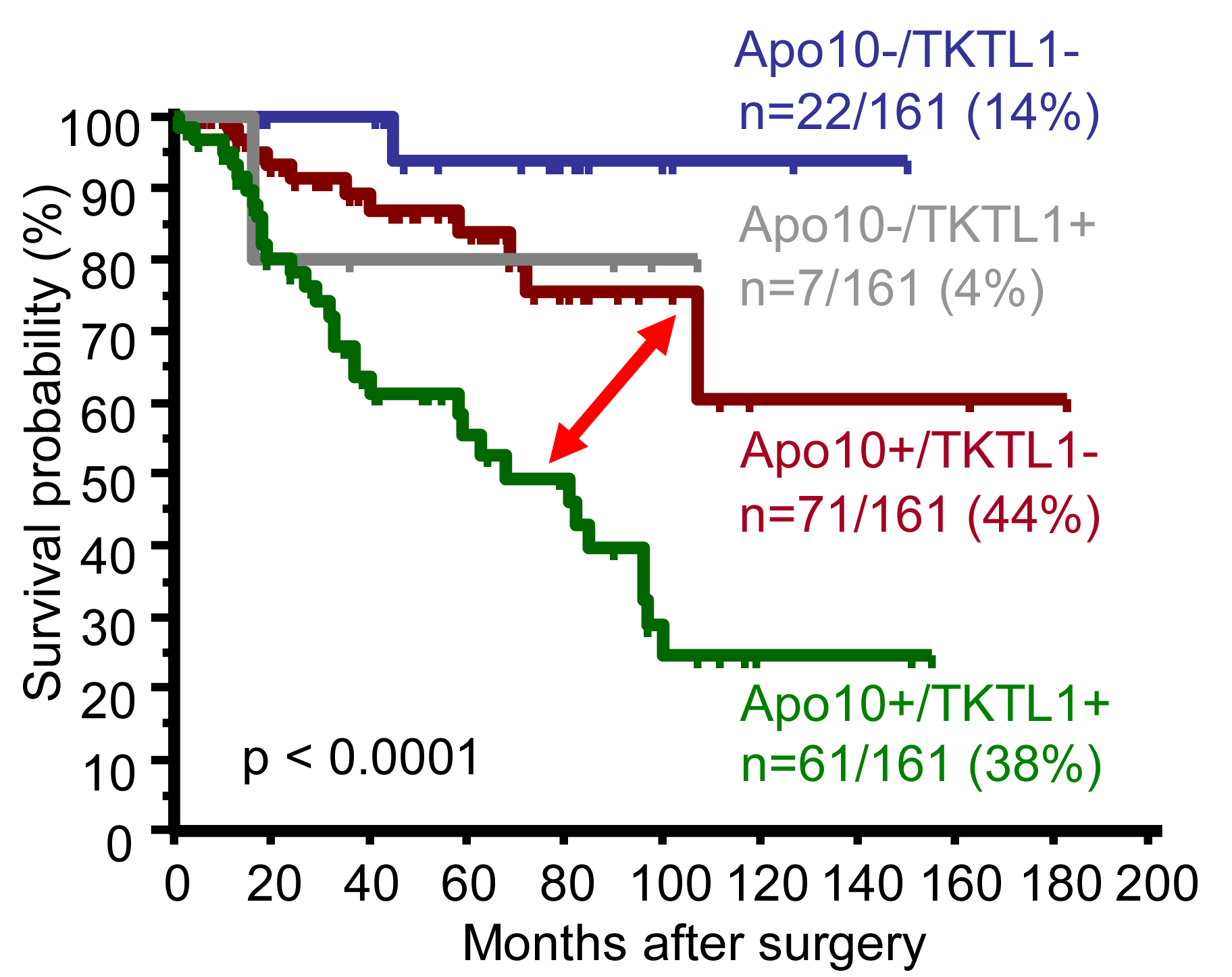

Supplement: Additional file 3 — Survival curve of OSCC patient subgroup analysis measured by Apo10/TKTL1 co-expression. Kaplan-Meier survival curves for DFS stratified by Apo10-/TKTL1- (blue line), Apo10+/TKTL1- (red line), Apo10-/TKTL1+ (grey line), and Apo10+/TKTL1+ (green line) subgroups (a). Compared with Apo10+/TKTL1- (red line), Apo10+/TKTL1+ (green line) subgroup shows the worst DFS (red arrow, p = 0.0002). The most favorable prognosis is demonstrated by the Apo10-/TKTL1- (blue line) subgroup. [file 1471-2407-13-569-S3.tiff]

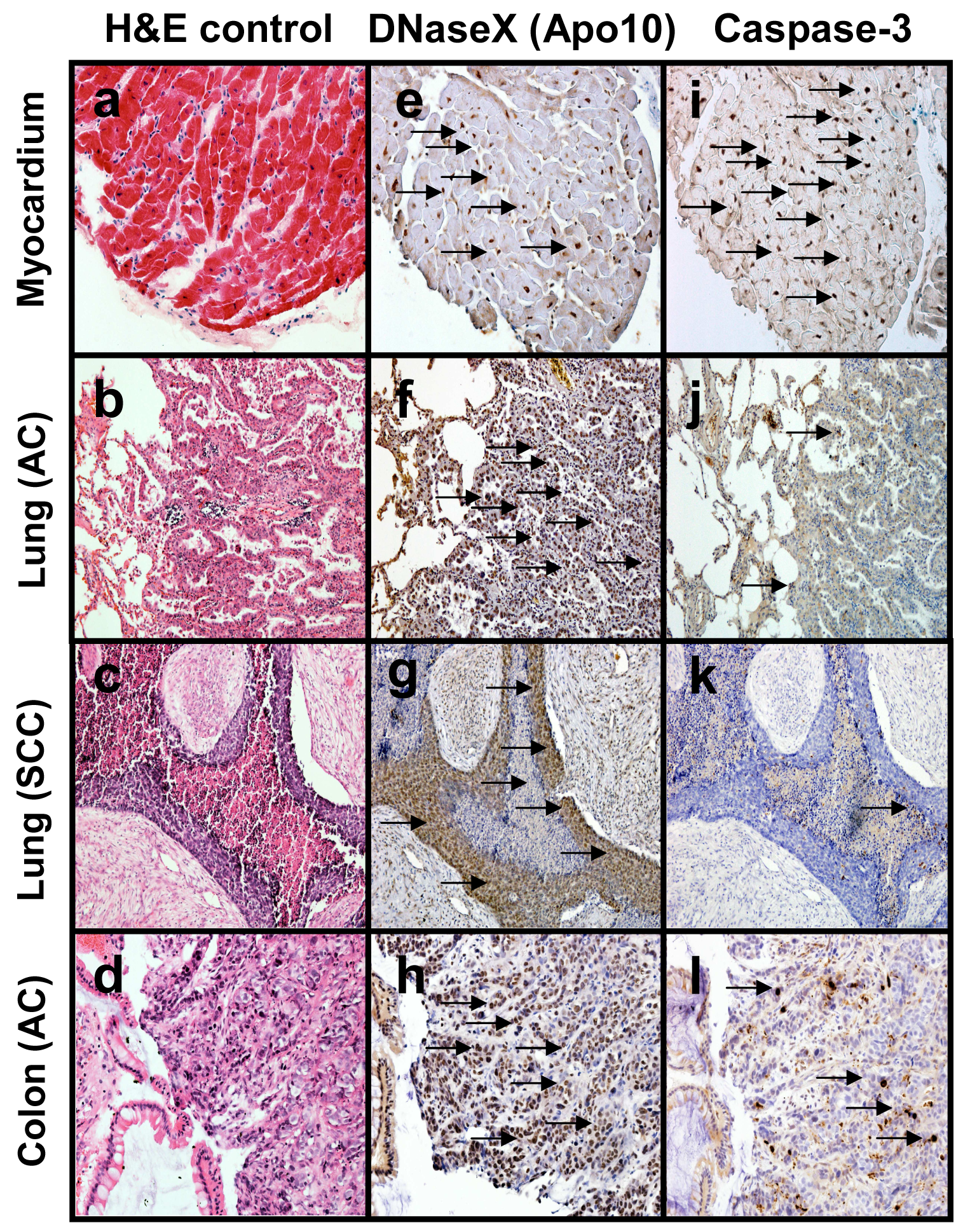

Supplement: Additional file 4 — DNaseX (Apo10) staining in benign cells of the myocardium and two different human epithelial tumor entities - carcinomas of the lung, and colon. Hematoxylin and eosin stain (H&E) shows myocardium (a) and different types of carcinomas (b, c, d). Immunohistochemistry shows representative images of Apo10 (e, f, g, h, arrows) in human apoptotic (Caspase-3 cleaved, i, j, k, l, arrows) benign cells of a patient after myocarditis and in carcinomas of the lung, and colon, which is detected in the nucleus. Apoptotic cells (Caspase-3 cleaved) are increased in benign tissue (i) compared with decreased detection of apoptotic cells in carcinomas (j, k, l). Both, benign and malign tissue types stained Apo10+. The blue color shows the nuclear counterstaining by hematoxylin. Original magnification: ×200-fold. AC, adenocarcinoma; SCC, Squamous cell carcinoma. [file 1471-2407-13-569-S4.tiff]

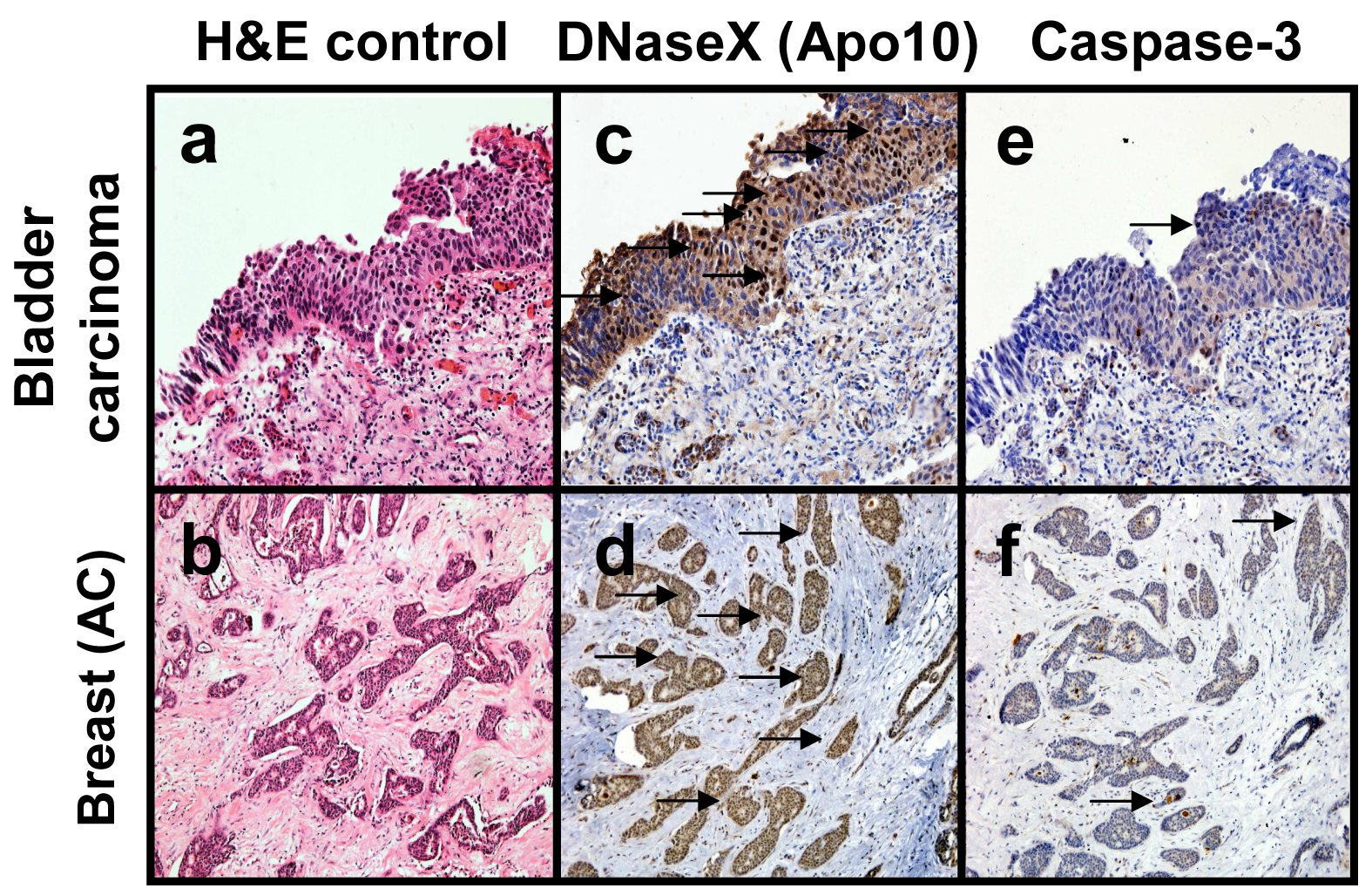

Supplement: Additional file 5 — DNaseX (Apo10) staining in bladder and breast carcinoma. Hematoxylin and eosin stain (H&E) shows bladder and breast carcinomas (a, b). Immunohistochemistry shows representative images of Apo10 (c, d, arrows) in human apoptotic (Caspase-3 cleaved, e, f, arrows) cells in carcinomas of the bladder and mammary gland (breast), which is detected in the nucleus. Apoptotic cells (Caspase-3 cleaved) in carcinomas (e, f) are decreased compared with benign tissue (myocardium, Additional file 4). Both, benign and malign tissue types stained Apo10+. The blue color shows the nuclear counterstaining by hematoxylin. Original magnification: ×200-fold. AC, adenocarcinoma. [file 1471-2407-13-569-S5.tiff]

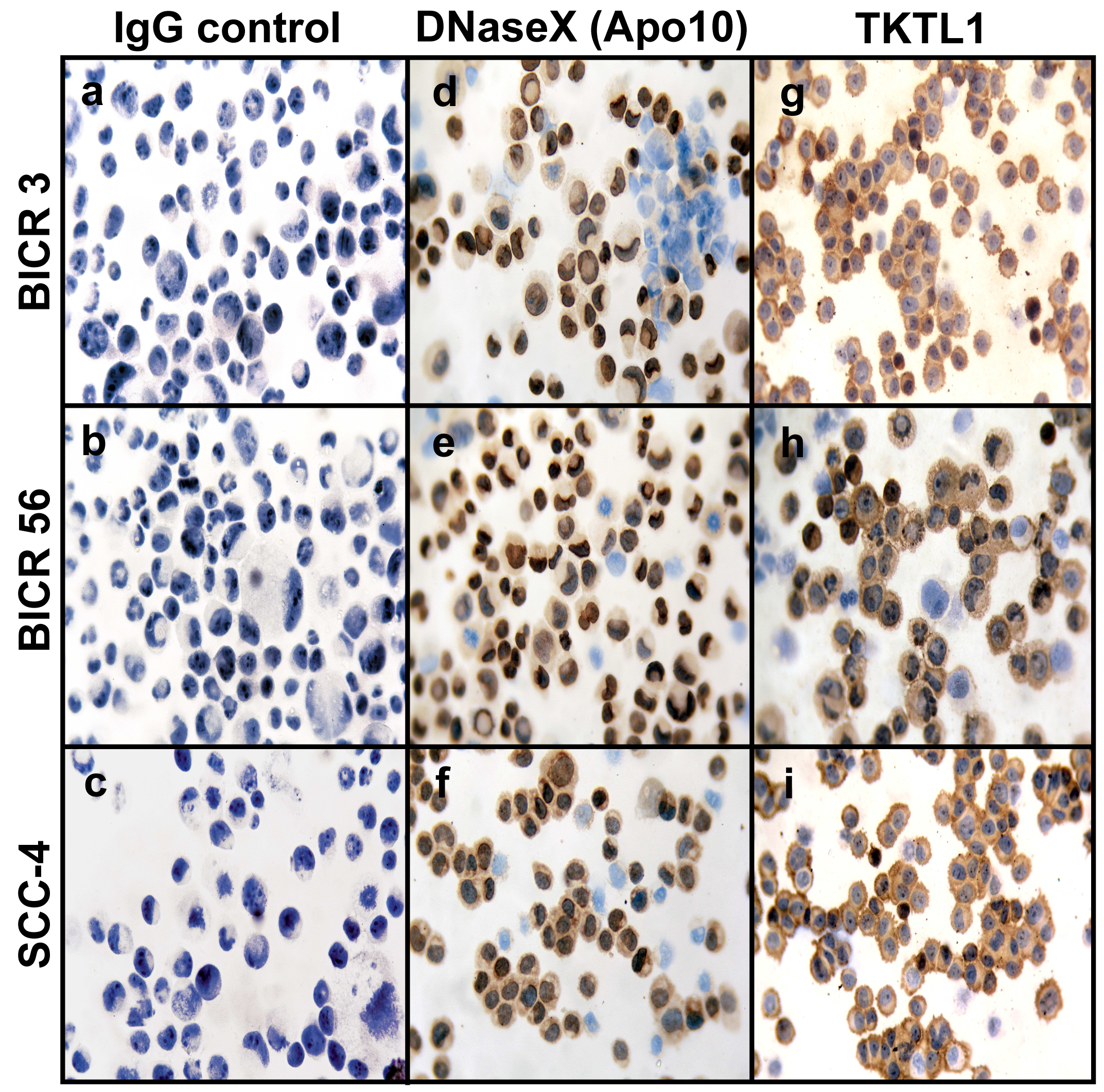

Supplement: Additional file 6 — DNaseX (Apo10) and TKTL1 immunocytochemical staining in BICR3, BICR56, and SCC-4 OSCC cell lines. IgG control shows no staining (a, b, c). Images show representative immunocytochemical staining of Apo10 (nuclear and weak cytoplasmic expression pattern, d, e, f), and TKTL1 (cytoplasmic staining expression pattern, g, h, i). The blue color shows the nuclear counterstaining by hematoxylin. Original magnification: ×400-fold. [file 1471-2407-13-569-S6.tiff]

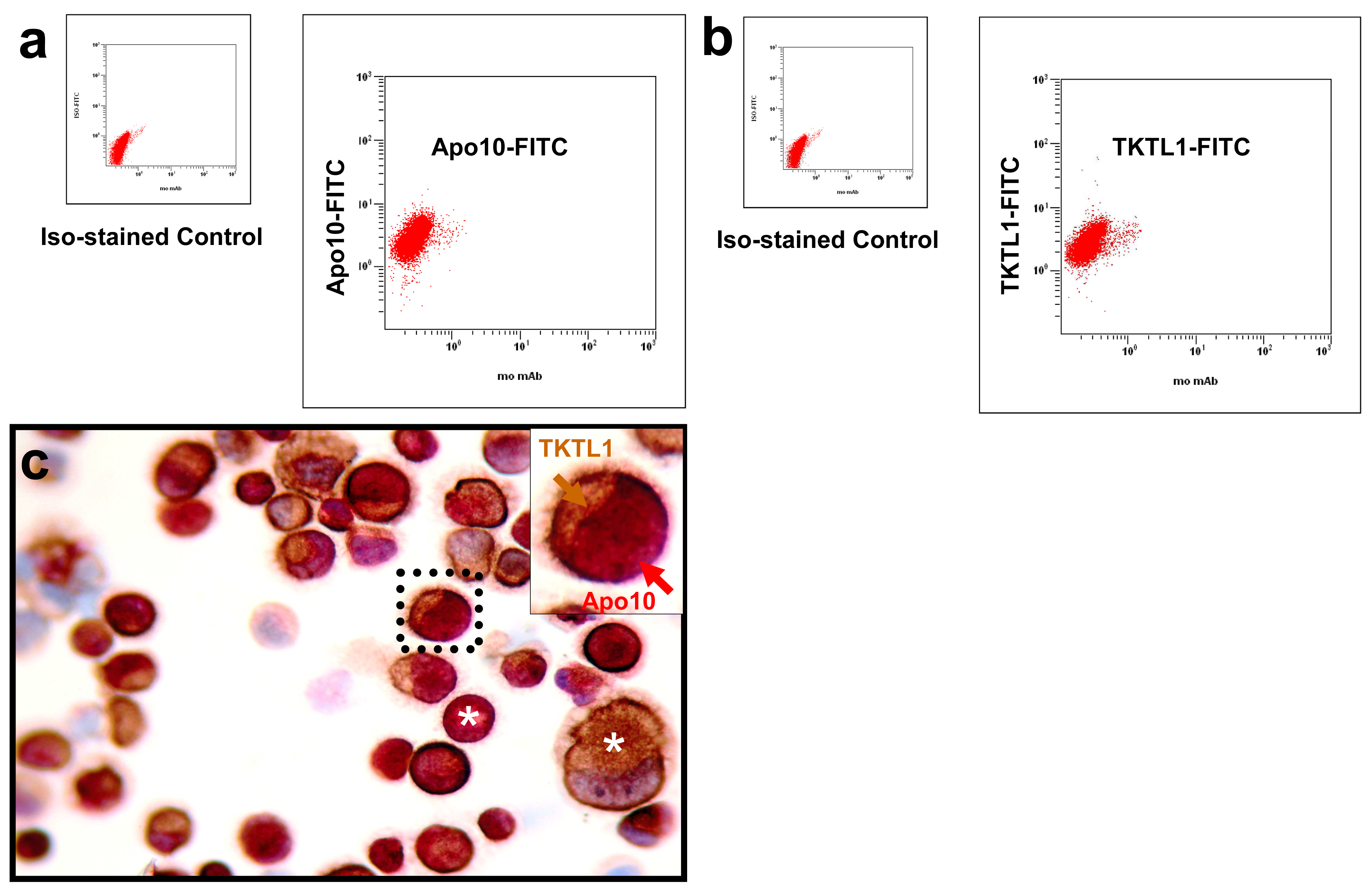

Supplement: Additional file 7 — Flow cytometric analysis of Apo10+, TKTL1+ cancer cells and immunocytochemical Apo10+/TKTL1+ double staining. Flow cytometric analysis shows representative Apo10 (a) and TKTL1 (b) labeling in BICR56 cancer cells as a positive control. FITC, Fluoresceinisothiocyanate. Immunocytochemical staining shows a representative image of Apo10+/TKTL1+ (c) tumor cells in BICR56 OSCC cell line. The red nuclear chromogen color (Fast Red) indicates positive Apo10 staining and the brown cytoplasmic chromogen color (DAB) indicates positive TKTL1 staining (arrows). Asterisks show single Apo10 (red) or TKTL1 (brown) positive cells. Original magnification: ×400-fold. [file 1471-2407-13-569-S7.tiff]

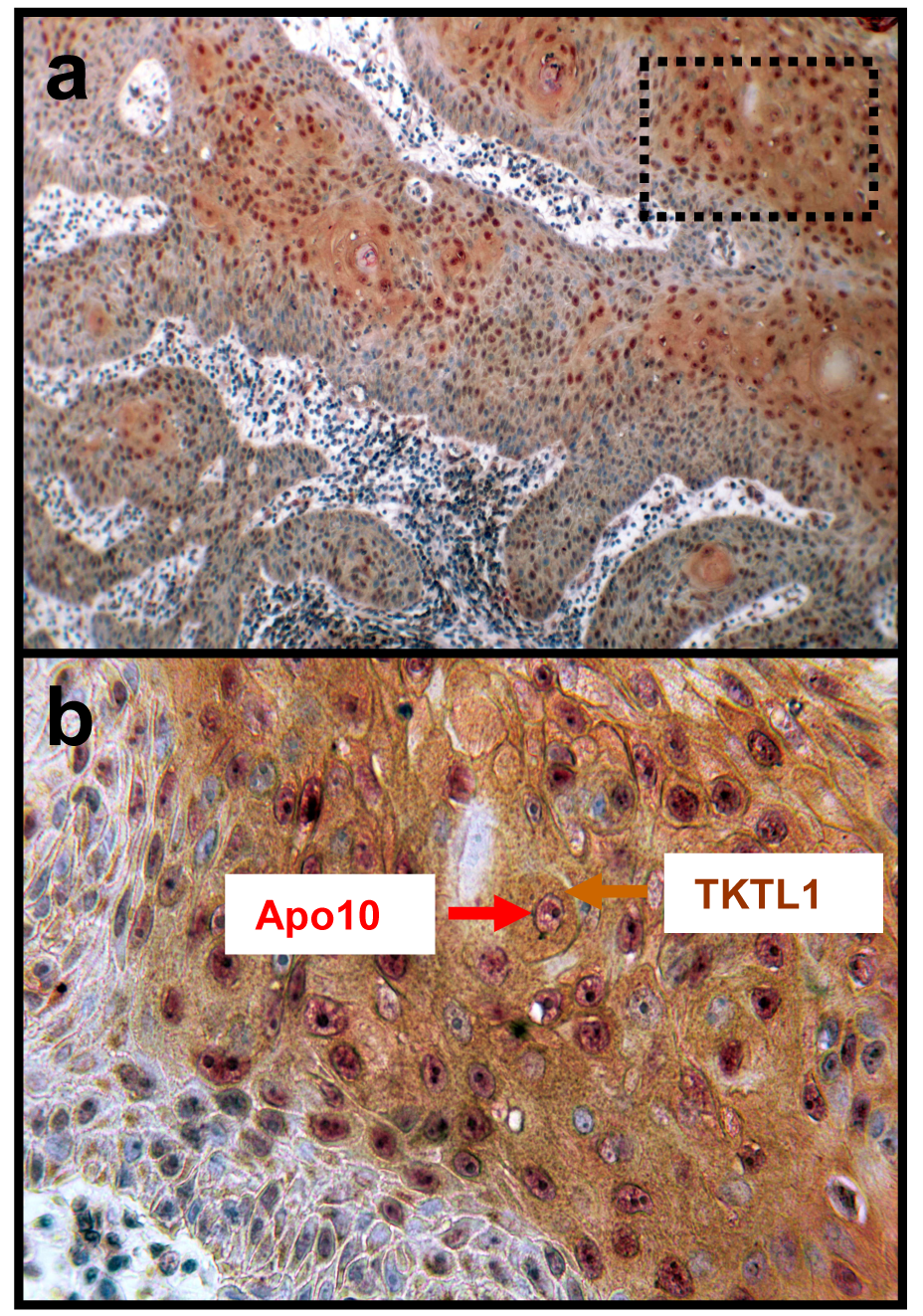

Supplement: Additional file 8 — Immunohistochemical Apo10+/TKTL1+ double staining. Immunohistochemical Apo10+/TKTL1+ double staining of a representative double positive OSCC tissue shows nuclear Apo10+ (red arrow, Fast Red) and cytoplasmic TKTL1+ (brown arrow, DAB) co-expression. The square box demonstrates area of interest (original magnification: ×100-fold, a), which is also shown in a larger magnification (×200-fold, lower panel, b). [file 1471-2407-13-569-S8.tiff]

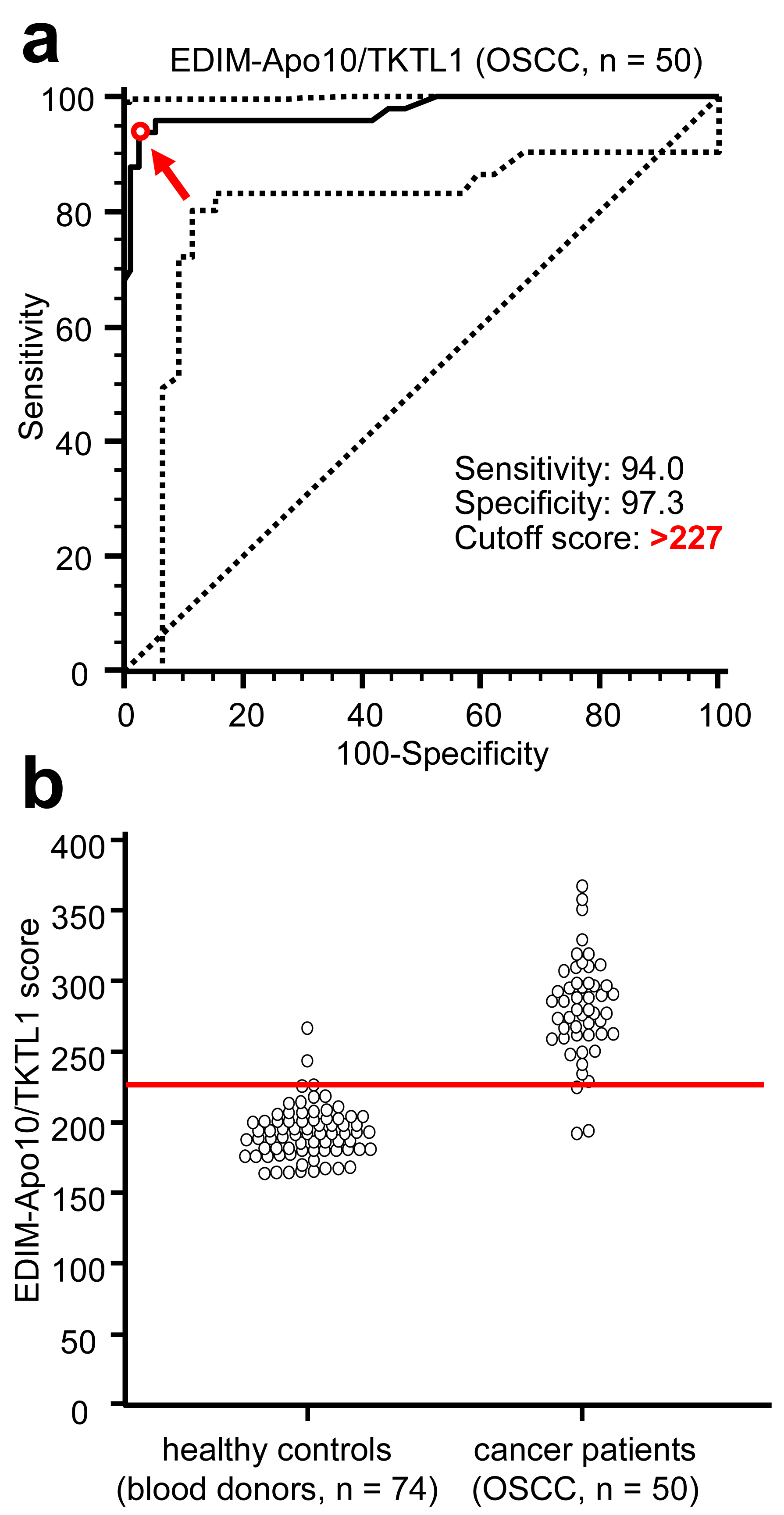

Supplement: Additional file 9 — Receiver Operating Characteristics (ROC) analysis of combined EDIM Apo10/TKTL1 score in OSCC (n = 50) compared with healthy individuals (n = 74), and interactive dot diagrams. The true positive rates (sensitivity) are plotted in function of the false positive rate (100-specificity) for measurement of the cut-off point: ROC analysis for the diagnosis of primary or recurrent OSCC shows calculated cut-off value with highest diagnostic accuracy (arrows) of combined EDIM Apo10/TKTL1 (a) score combined EDIM-Apo10 plus EDIM-TKTL1 score >227: sensitivity 94.0%, 95% CI 83.5–98.7%, specificity 97.3%, 95% CI 90.6–99.7%). Dotted lines show 95% CI. OSCC, oral squamous cell carcinoma. In the interactive dot diagrams (part of ROC curve analysis, b) the data of healthy controls and OSCC group are displayed as dots on two vertical axes. The horizontal line indicates the cut-off points with the best separation/highest accuracy (minimal false negative and false positive results) between healthy controls and OSCC group. The corresponding test characteristics sensitivity and specificity are shown above. [file 1471-2407-13-569-S9.tiff]

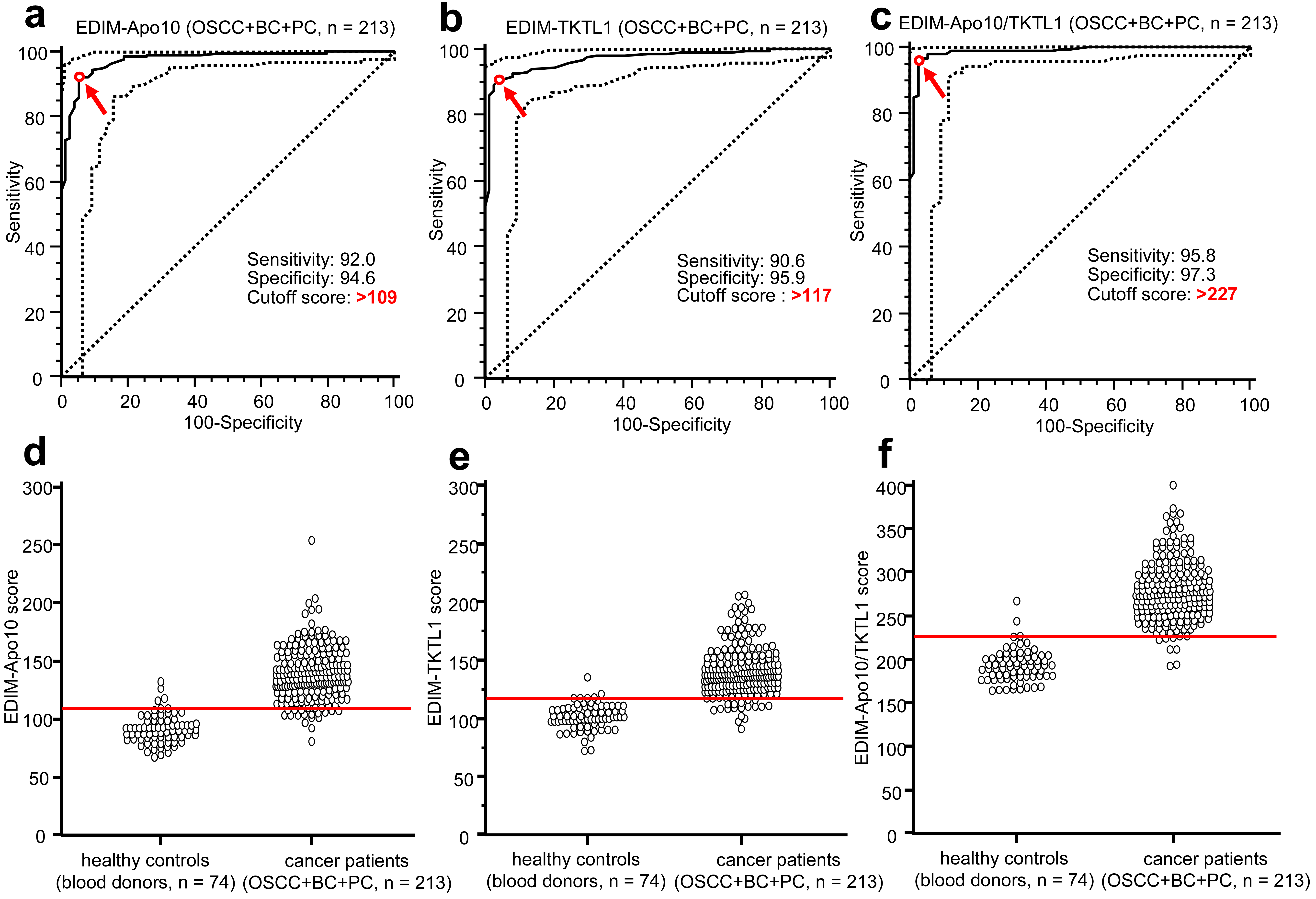

Supplement: Additional file 13 — Receiver Operating Characteristics (ROC) analysis of EDIM-Apo10, EDIM-TKTL1, and combined EDIM Apo10/TKTL1 score in all cancer samples (OSCC, breast and prostate cancer, n = 213) compared with healthy individuals (n = 74). The true positive rates (sensitivity) are plotted in function of the false positive rate (100-specificity) for measurement of the cut-off point: ROC analysis for the diagnosis of all cancer samples/entities (OSCC, breast and prostate cancer, a-c) shows calculated cut-off value with highest diagnostic accuracy (arrows) of EDIM-Apo10 (a), EDIM-TKTL1 (b), and combined EDIM Apo10/TKTL1 (c) score (a, EDIM-Apo10 score >109: sensitivity 92.0%, 95% CI 87.5–95.3%, specificity 94.6%, 95% CI 86.7–98.5%; b, EDIM-TKTL1 score >117: sensitivity 90.6%, 95% CI 85.9–94.2%, specificity 95.9%, 95% CI 88.6–99.2%; c, combined EDIM-Apo10 plus EDIM-TKTL1 score >227: sensitivity 95.8%, 95% CI 92.1–98.0%, specificity 97.3%, 95% CI 90.6–99.7%). Dotted lines show 95% CI. OSCC, oral squamous cell carcinoma; BC, breast cancer; PC, prostate cancer. In the interactive dot diagrams (part of ROC curve analysis, d-f) the data of healthy controls and cancer group are displayed as dots on two vertical axes. The horizontal line indicates the cut-off points with the best separation/highest accuracy (minimal false negative and false positive results) between healthy controls and cancer group. The corresponding test characteristics sensitivity and specificity are shown above. [file 1471-2407-13-569-S13.tiff]
