# Supplementary material for: A biomarker based detection and characterization of carcinomas exploiting two fundamental biophysical mechanisms in mammalian cells
Source: BMC Cancer. 2013 Dec 4;13:569. doi: 10.1186/1471-2407-13-569 (PMC4235042; doi:10.1186/1471-2407-13-569)
Supplement: Additional file 10: Table S1 — Pre- and postoperative epitope detection in monocytes (EDIM)-Apo10 and TKTL1 scores in patients with oral squamous cell carcinoma (n = 3). [file 1471-2407-13-569-S10.doc]

**Table S**1 - pre- and postoperative epitope detection in monocytes (EDIM)-Apo10 and TKTL1 scores in patients with oral squamous cell carcinoma (n = 3)

| **Characteristics** | **preoperative** | | | **postoperative** | |
| --- | --- | --- | --- | --- | --- |
|  | Total  n=3 | Apo10 score | TKTL1 score | Apo10 score | TKTL1 score |
| Patient 1 |  | 143 | 134 | 102 | 111 |
| Patient 2 |  | 119 | 146 | 99 | 102 |
| Patient 3 |  | 124 | 121 | 100 | 93 |
