# Supplementary material for: A biomarker based detection and characterization of carcinomas exploiting two fundamental biophysical mechanisms in mammalian cells
Source: BMC Cancer. 2013 Dec 4;13:569. doi: 10.1186/1471-2407-13-569 (PMC4235042; doi:10.1186/1471-2407-13-569)
Supplement: Additional file 11: Table S2 — Pre- and postoperative epitope detection in monocytes (EDIM)-Apo10 and TKTL1 scores in patients with breast cancer (n = 3). [file 1471-2407-13-569-S11.doc]

### Table S2 - pre- and postoperative epitope detection in monocytes (EDIM)-Apo10 and TKTL1 scores in patients with breast cancer (n = 3)

| **Characteristics** | **preoperative** | | | **postoperative** | |
| --- | --- | --- | --- | --- | --- |
|  | Total  n=3 | Apo10 score | TKTL1 score | Apo10 score | TKTL1 score |
| Patient 1 |  | 161 | 129 | 81 | 100 |
| Patient 2 |  | 126 | 155 | 96 | 77 |
| Patient 3 |  | 133 | 132 | 98 | 89 |
