# Supplementary material for: A biomarker based detection and characterization of carcinomas exploiting two fundamental biophysical mechanisms in mammalian cells
Source: BMC Cancer. 2013 Dec 4;13:569. doi: 10.1186/1471-2407-13-569 (PMC4235042; doi:10.1186/1471-2407-13-569)
Supplement: Additional file 12: Table S3 — Pre- and postoperative epitope detection in monocytes (EDIM)-Apo10 and TKTL1 scores in patients with prostate cancer (n = 6). [file 1471-2407-13-569-S12.doc]

### Table S3 - pre- and postoperative epitope detection in monocytes (EDIM)-Apo10 and TKTL1 scores in patients with prostate cancer (n = 6)

| **Characteristics** | **preoperative** | | | **postoperative** | |
| --- | --- | --- | --- | --- | --- |
|  | Total  n=6 | Apo10 score | TKTL1 score | Apo10 score | TKTL1 score |
| Patient 1 |  | 155 | 149 | 98 | 99 |
| Patient 2 |  | 144 | 166 | 93 | 106 |
| Patient 3 |  | 153 | 165 | 93 | 101 |
| Patient 4 |  | 162 | 143 | 105 | 104 |
| Patient 5 |  | 139 | 149 | 102 | 88 |
| Patient 6 |  | 158 | 144 | 95 | 95 |
