# Supplementary material for: A biomarker based detection and characterization of carcinomas exploiting two fundamental biophysical mechanisms in mammalian cells
Source: BMC Cancer. 2013 Dec 4;13:569. doi: 10.1186/1471-2407-13-569 (PMC4235042; doi:10.1186/1471-2407-13-569)
Supplement: Additional file 14: Table S4 — Adjuvant treatment of 161 patients with OSCC according to UICC stages. [file 1471-2407-13-569-S14.doc]

### Table S4 – Adjuvant treatment of 161 patients with OSCC according to UICC stages.

| **Characteristics** | **Adjuvant treatment (RTx, CTx)** | | | **p-value** |
| --- | --- | --- | --- | --- |
|  | Total  n=161 | no  n=111 (69%) | yes  n=50 (31%) |  |
| UICC stage |  |  |  | < 0.0001 |
| UICC I/II | 84 (52.2%) | 82 (98%) | 2 (2%) |  |
| UICC III/IV | 77 (47.8%) | 29 (38%) | 48 (62%) |  |
| RTx, radiotherapy; CTx, chemotherapy | | | |  |
